# CCL2-mediated inflammatory pathogenesis underlies high myopia-related anxiety

**DOI:** 10.1038/s41421-023-00588-2

**Published:** 2023-09-12

**Authors:** Xiangjia Zhu, Jiaqi Meng, Chaofeng Han, Qingfeng Wu, Yu Du, Jiao Qi, Ling Wei, Hao Li, Wenwen He, Keke Zhang, Yi Lu

**Affiliations:** 1 Department of Ophthalmology, Eye & ENT Hospital, Fudan University, Shanghai, China; 2 NHC Key Laboratory of Myopia, Fudan University, Shanghai, China; 3 Key Laboratory of Myopia, Chinese Academy of Medical Sciences, Shanghai, China; 4 Shanghai Key Laboratory of Visual Impairment and Restoration, Shanghai, China; 5 State Key Laboratory of Medical Neurobiology, Fudan University, Shanghai, China; 6 Department of Histoembryology, Naval Medical University, Shanghai, China; 7 Shanghai Key Laboratory of Cell Engineering, Naval Medical University, Shanghai, China; 8 State Key Laboratory of Molecular Development Biology, Chinese Academy of Sciences, Beijing, China; 9 Institute of Genetics and Developmental Biology, University of Chinese Academy of Sciences, Beijing, China; 10 CAS Center for Excellence in Brain Science and Intelligence Technology, Shanghai, China; 11 Chinese Institute for Brain Research, Beijing, China

**Keywords:** Innate immunity, Mechanisms of disease

## Abstract

High myopia is a leading cause of blindness worldwide. It may lead to emotional defects that rely closely on the link between visual sensation and the central nervous system. However, the extent of the defects and its underlying mechanism remain unknown. Here, we report that highly myopic patients exhibit greater anxiety, accompanied by higher CC chemokine ligand 2 (CCL2) and monocyte levels in the blood. Similar findings are found in the mouse model of high myopia. Mechanistic evaluations using GFP-positive bone marrow chimeric mice, parabiotic mouse model, enhanced magnetic resonance imaging, etc., show that highly myopic visual stimulation increases CCL2 expression in eyes, aggravates monocyte/macrophage infiltration into eyes and brains, and disrupts blood–ocular barrier and blood–brain barrier of mice. Conversely, *Ccl2*-deficient highly myopic mice exhibit attenuated ocular and brain infiltration of monocytes/macrophages, reduced disruption of the blood–ocular barrier and blood–brain barrier, and less anxiety. Substantial alleviation of high myopia-related anxiety can also be achieved with the administration of CCL2-neutralizing antibodies. Our results establish the association between high myopia and anxiety, and implicate the CCL2-mediated inflammatory pathogenesis as an underlying mechanism.

## Introduction

High myopia, defined as eyes with an axial length (AL) ≥ 26 mm, is a leading cause of blindness worldwide^1^. It is a risk factor for many severe ocular complications, including macular degeneration, cataract, and optic neuropathy^2–4^. Another interesting phenomenon that all ophthalmologists think exists, but lacks systematic investigations is that patients with high myopia tend to suffer from greater anxiety and usually require repeated confirmation of their ocular condition by their ophthalmologists, compounding the difficulty of the treatment and greatly impairing their quality of life^5–7^. However, the anxiety associated with high myopia remains merely a clinical impression that has not been validated yet by in-depth research, let alone the underlying mechanisms.

Recent studies have shown that CC chemokine ligand 2 (CCL2), which regulates the recruitment of peripheral mononuclear cells, plays a critical role in anxiety-related psychiatric disorders, such as social defeat stress, alcohol-withdrawal-induced anxiety, and suicidal tendencies^8–10^. Increased CCL2 has been detected in the aqueous humor of highly myopic eyes and is associated with the stage of myopic maculopathy^11,12^, suggesting a CCL2-related inflammatory pathogenesis. Therefore, we speculated that CCL2 might also play an essential role in high myopia-related anxiety via inflammatory pathogenesis. However, relevant evidence remains sparse.

The regulation of monocytes/macrophages by CCL2 and its augmentation of inflammation also involve the disruption of immune barriers^13,14^. Evidence has suggested a role of blood–brain barrier (BBB) disruption in CCL2-related psychiatric disorders, including anxiety, depression, and social avoidance^15–18^. Thus, regarding high myopia-related anxiety, the disruption of the blood–ocular barrier (BOB) and the BBB may also be involved in the CCL2-mediated inflammation in the eyes and the defective emotional responses in the brain.

To corroborate our hypothesis, we first verified the increased anxiety level in highly myopic patients using the Hamilton Anxiety Scale (HAMA), which was positively correlated with the CCL2 and monocyte levels in their blood. Similar findings were recapitulated in the mouse model of high myopia. Mechanistic explorations using GFP-positive bone marrow chimeric mice, parabiotic mouse model, enhanced magnetic resonance imaging (MRI), etc., revealed that highly myopic visual stimulation upregulated CCL2 expression in eyes, increased the infiltration of monocytes/macrophages in eyes and brains, and disrupted the BOB/BBB. Further investigations with *Ccl2-*deficient mice and mice treated with CCL2-neutralizing antibodies showed that high myopia-related anxiety could be substantially attenuated by the inhibition of CCL2.

## Results

### Highly myopic patients exhibit greater anxiety and higher CCL2 and monocyte levels in the blood

**Fig. 1:**
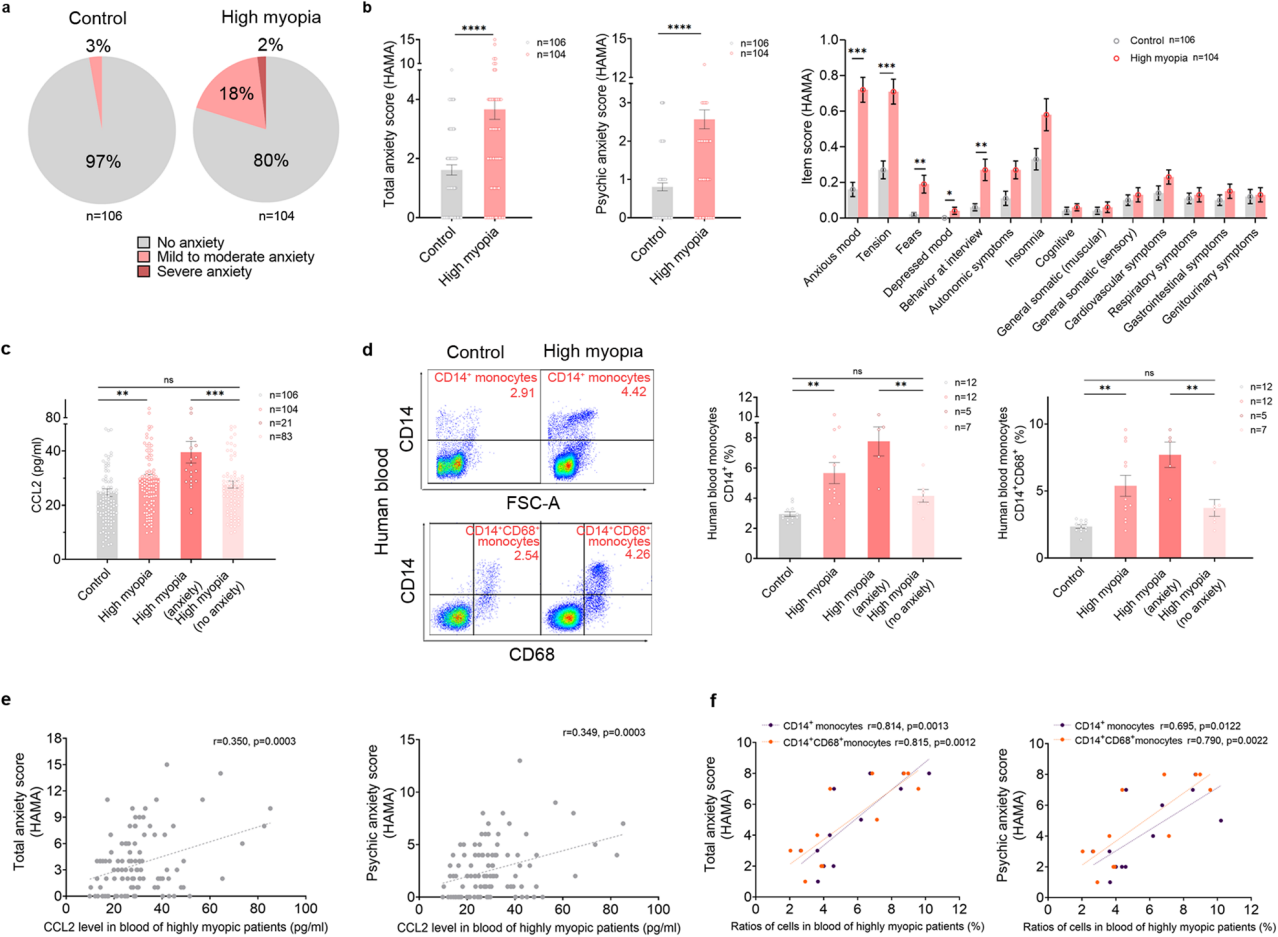
Highly myopic patients exhibit higher anxiety scores and elevated CCL2 and monocyte levels in the blood. **a** Proportions of no anxiety, mild to moderate anxiety, and severe anxiety in the control and highly myopic groups. **b** Total anxiety scores, psychic anxiety scores, and item scores on the HAMA. **c** Measurement of CCL2 concentration in human blood with a cytokine array. *n* = 106 in the control group, *n* = 104 in the highly myopic group, *n* = 21 in the highly myopic group with anxiety (total anxiety score ≥ 7), and *n* = 83 in the highly myopic group with no anxiety (**a**–**c**). **d** Flow-cytometric quantification of monocytes (CD14^+^) and CD14^+^/CD68^+^ monocytes in human blood. *n* = 12 in the control and highly myopic groups, *n* = 5 in the highly myopic group with anxiety, and *n* = 7 in the highly myopic group with no anxiety. **e** Correlations between total and psychic anxiety scores and CCL2 levels in the blood of highly myopic patients (*n* = 104). **f** Correlations between total and psychic anxiety scores and ratios of monocytes (CD14^+^) and CD14^+^/CD68^+^ monocytes in the blood of highly myopic patients (*n* = 12). Data are mean ± SD. Level of significance was detected with χ^2^ test (**a**), Mann–Whitney *U* test (**b**), Student’s *t-*test (**c**, **d**), and Pearson’s analysis (**e**, **f**). ****P < 0.0001, ***P < 0.001, **P < 0.01, ns *P* > 0.05.

To evaluate the anxiety levels of highly myopic patients, we collected HAMA questionnaires from 104 highly myopic patients and 106 emmetropic controls. There were no significant differences in age, sex, body mass index, smoking, hypertension, alcohol consumption, occupation type, and educational degree between the two groups (Supplementary Table S1). In the highly myopic group, 18% had mild to moderate anxiety and 2% had severe anxiety; whereas in the control group, the percentages were only 3% and 0%, respectively (*P* < 0.001 and *P* = 0.001, respectively; Fig. 1a). Overall, highly myopic patients displayed significantly higher total anxiety scores (3.66 ± 3.43 vs 1.61 ± 1.77, respectively, *P* < 0.0001) and psychic anxiety scores (2.57 ± 2.51 vs 0.88 ± 1.22, respectively, *P* < 0.0001) (Fig. 1b). Anxious mood, tension, fear, depressed mood, and behavior at the interview were the main psychic anxiety indicators found to be increased in the highly myopic group (all *P* < 0.05; Fig. 1b).

In our previous study, we identified elevated CCL2 levels in the aqueous humor of highly myopic eyes^11^. To further explore the role of CCL2 in the anxiety of high myopes, we analyzed human blood samples with a suspension cytokine array and identified significantly higher levels of CCL2 in the blood of highly myopic patients (30.04 ± 14.19 pg/mL vs 24.64 ± 14.63 pg/mL in the control, *P* = 0.0073; Fig. 1c). Furthermore, among highly myopic patients, those with anxiety (total anxiety score ≥ 7) displayed significantly higher CCL2 levels than those with no anxiety (39.59 ± 18.36 pg/mL vs 27.62 ± 11.90 pg/mL, respectively, *P* = 0.0004; Fig. 1c), suggesting that highly myopic patients with higher CCL2 levels tend to have a higher risk of anxiety.

Because CCL2 is an important chemokine that is known to regulate monocytes, we also quantified the monocyte levels in human blood with flow cytometry. The highly myopic patients showed significantly elevated ratios of monocytes (CD14^+^; 5.66 ± 2.41% vs 2.94 ± 0.52% in the control, *P* = 0.0024) and CD14^+^/CD68^+^ monocytes (5.39 ± 2.70% vs 2.35 ± 0.45% in the control, *P* = 0.0024) in the blood (Fig. 1d). Furthermore, among the highly myopic patients, those with anxiety (total anxiety score ≥ 7) also had higher ratios of monocytes (7.77 ± 2.15% vs 4.16 ± 1.10%, respectively, *P* = 0.0032) and CD14^+^/CD68^+^ monocytes (7.71 ± 2.12% vs 3.74% ± 1.65%, respectively, *P* = 0.0064) than those with no anxiety (Fig. 1d).

Both the total and psychic anxiety scores correlated positively with the CCL2 and monocyte levels in the blood of highly myopic patients (CCL2: *r* = 0.350 and 0.349, respectively, both *P* = 0.0003; Fig. 1e; monocytes: *r* = 0.814 and 0.695, respectively, both *P* < 0.05; CD14^+^/CD68^+^ monocytes: *r* = 0.815 and 0.790, respectively, both *P* < 0.01; Fig. 1f).

The levels of inflammatory cytokines, including MIP-1β, RANTES, and TNF-α, were also significantly higher in the blood of the highly myopic patients. Among the highly myopic patients, the levels of MIP-1β and TNF-α were significantly higher in those with anxiety than those with no anxiety (all *P* < 0.05; Supplementary Fig. S1).

### The highly myopic mouse model exhibits increased anxiety-like behaviors, upregulated CCL2 expression in the eyes, and higher CCL2 and monocyte levels in the blood

**Fig. 2:**
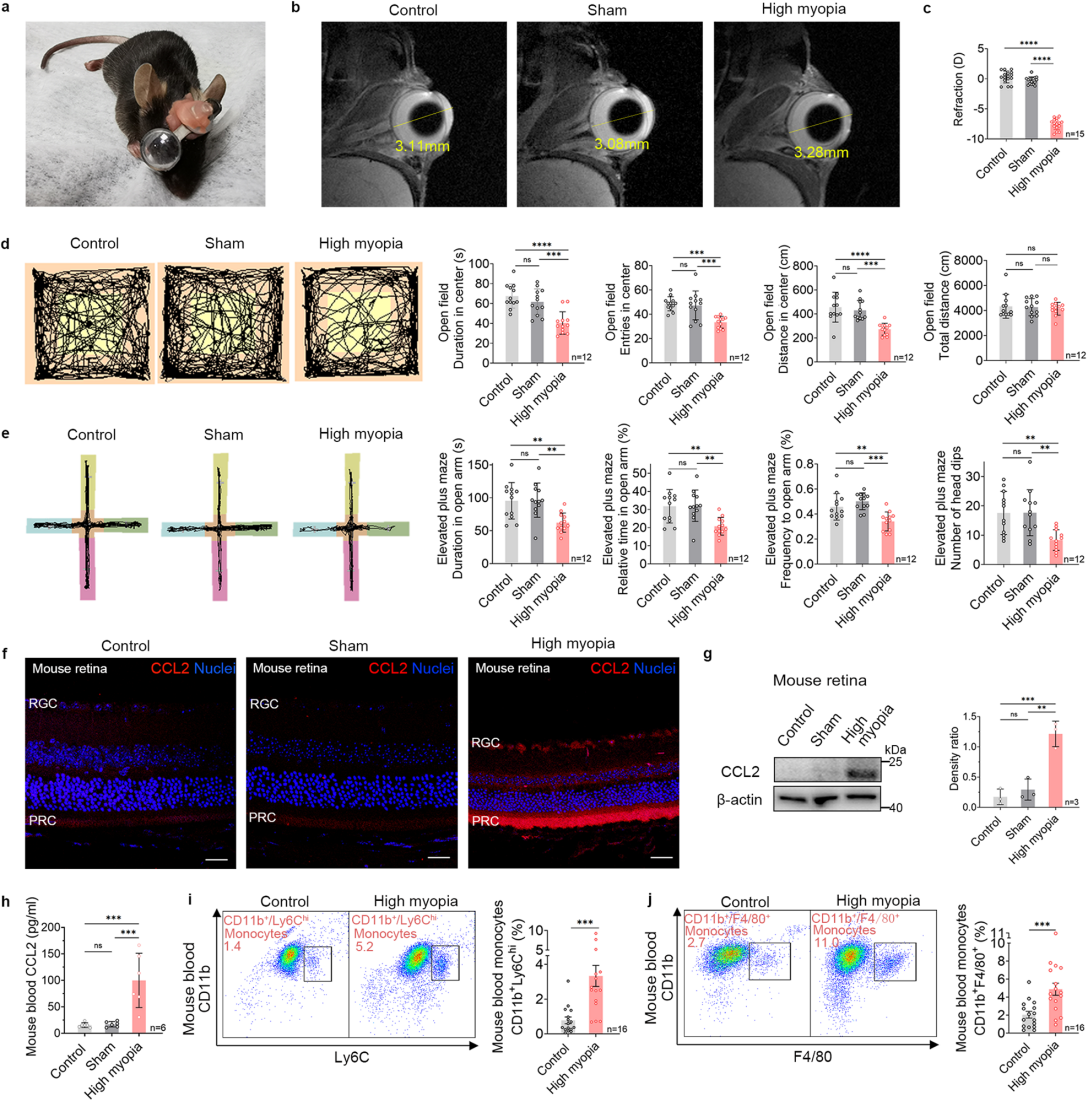
Highly myopic mouse model exhibits increased anxiety-like behaviors, upregulated CCL2 expression in the eye, and higher CCL2 and monocyte levels in the blood. **a** Representative photograph of a mouse wearing a −10 D lens to induce high myopia in the right eye. **b** Representative images of ocular MRI (sagittal) showing an obviously longer AL in the highly myopic eye than in the control and sham eyes. Yellow lines indicate the measurement of AL. **c** The refraction of the control, sham, and highly myopic eyes (*n* = 15 in each group). **d** Representative maps of animal tracking (black lines) in the dark showing increased anxiety-like behaviors in the mouse model of high myopia, manifested as shorter duration, fewer entries, and shorter distance traveled in the center (yellow square) of the open field (*n* = 12 in each group). **e** Representative maps of animal tracking (black lines) in the dark showing increased anxiety-like behaviors in the mouse model of high myopia, manifested as shorter duration, fewer entries, and fewer head dips in the open arms (pink and yellow area) of the elevated plus maze (*n* = 12 in each group). The blue and green areas refer to closed arms and the orange area refers to the center of the maze. **f** Immunofluorescent images of CCL2 staining in the mouse retina showing upregulated CCL2 expression in the RGC layer and PRC layer of highly myopic mice (*n* = 3 in each group). Scale bars: 25 μm. **g** Examination of CCL2 expression in mouse retina with western blotting. The band density of CCL2 was normalized to the loading control for statistical analysis (*n* = 3 in each group). **h** Measurement of CCL2 concentration in mouse blood with a proinflammatory biomarker array (*n* = 6 in each group). **i**, **j** Flow-cytometric quantification showed higher ratios of CD11b^+^/Ly6C^hi^ monocytes and CD11b^+^/F4/80^+^ monocytes in the blood of highly myopic mice than in the controls (*n* = 16 in each group). Data are mean ± SD. Levels of significance were detected with one-way ANOVA (**c**–**e**, **g**, **h**) and Student’s *t-*test (**i**, **j**). ****P < 0.0001, ***P < 0.001, **P < 0.01, *P < 0.05, ns *P* > 0.05.

To corroborate our findings in highly myopic patients, we established a highly myopic mouse model by attaching a −10 D lens over one eye in mice for 4 weeks (Fig. 2a). Mice wearing a 0 D plano lens were used as the sham group, and mice wearing no lens were used as the control group. As demonstrated with ocular MRI and refraction, eyes wearing a −10 D lens showed significantly longer AL and a myopic shift compared with both the control and sham eyes (Fig. 2b, c), suggesting the successful establishment of the mouse model of high myopia.

Behavioral assessment of anxiety using the open field test (OFT) and elevated plus maze (EPM) showed that the highly myopic mice displayed more anxiety-like behaviors than the control and sham mice, manifesting as shorter duration, fewer entries, and shorter distance traveling in the center of the open field as well as shorter duration, fewer entries, and fewer head dips in the open arms of the plus maze, in both light and dark conditions (Fig. 2d, e; Supplementary Fig. S2a). Furthermore, wearing a lens for myopic correction during behavioral tests did not reduce these anxiety-like behaviors in highly myopic mice (Supplementary Fig. S2b). No difference in total distance during OFT among the groups indicated their comparable baseline movement ability. To further rule out the effect of myopia-induced vision impairment on behavioral assessment, the Vogel test was also performed and the results confirmed that the highly myopic mice displayed more anxiety-like behaviors, including shorter drinking time, fewer licks and fewer shocks accepted than the control mice, while no difference in latency to drinking was found between the groups (Supplementary Fig. S2c). These data suggest that the highly myopic mouse model exhibits a higher risk of anxiety, corroborating our findings in humans. The anxiety was not reduced one week after the removal of the –10 D lens when vision was recovered (Supplementary Fig. S3a).

To evaluate the expression of CCL2 in eyes, we performed immunofluorescent staining and western blotting on mouse retina tissues and found increased expression of CCL2 in the retinal ganglion cell (RGC) layer and photoreceptor cell (PRC) layer of the highly myopic eyes (Fig. 2f, g). After recovery from high myopia, upregulated CCL2 in highly myopic eyes was reversed in mice (Supplementary Fig. S3b). These data suggest that sustained highly myopic visual stimulation increases the CCL2 level in ocular tissues.

We then used a proinflammatory biomarker array to screen for multiple inflammatory cytokines in the blood of these mice and identified higher CCL2 level in the highly myopic mice (high myopia vs control: 99.98 ± 51.29 pg/mL vs 15.47 ± 4.57 pg/mL, respectively, *P* = 0.0005; high myopia vs sham: 99.98 ± 51.29 pg/mL vs 17.19 ± 5.09 pg/mL, respectively, *P* = 0.0006) which was not reversed after recovery of high myopia (Supplementary Fig. S3c), whereas there was no difference between the control and sham groups (Fig. 2h). The duration in the center of the open field was negatively correlated with the CCL2 level in the blood of the highly myopic mice (*r* = –0.876, *P* = 0.0221; Supplementary Fig. S4a), suggesting that higher anxiety level is associated with higher blood CCL2 level in mice.

We also quantified the monocyte levels in the blood of these mice. We found a significantly higher ratio of CD11b^+^/Ly6C^hi^ monocytes, the inflammatory monocytes that can exit the bloodstream, patrol extravascular tissues, and differentiate into macrophages during the inflammatory response^19^ (3.33 ± 2.42% vs 0.77 ± 0.77% in the control, *P* = 0.0008), and CD11b^+^/F4/80^+^ monocytes (4.88 ± 2.70% vs 2.05 ± 1.50% in the control, *P* = 0.0009) in the blood of highly myopic mice (Fig. 2i, j), suggesting the boosted release of monocytes into the blood. These results indicate that sustained highly myopic visual stimulation activates the mononuclear phagocyte system in the circulation system, which may be mediated by the upregulation of CCL2.

We also identified significantly elevated levels of five inflammatory cytokines (MIP-1β, RANTES, TNF-α, IFN-γ, and IL-6) in the blood of highly myopic mice compared with those in the control and sham groups (Supplementary Fig. S4b–f).

### Elevated CCL2 expression increases the infiltration of blood-derived monocytes/macrophages in the eyes and brains of highly myopic mice

**Fig. 3:**
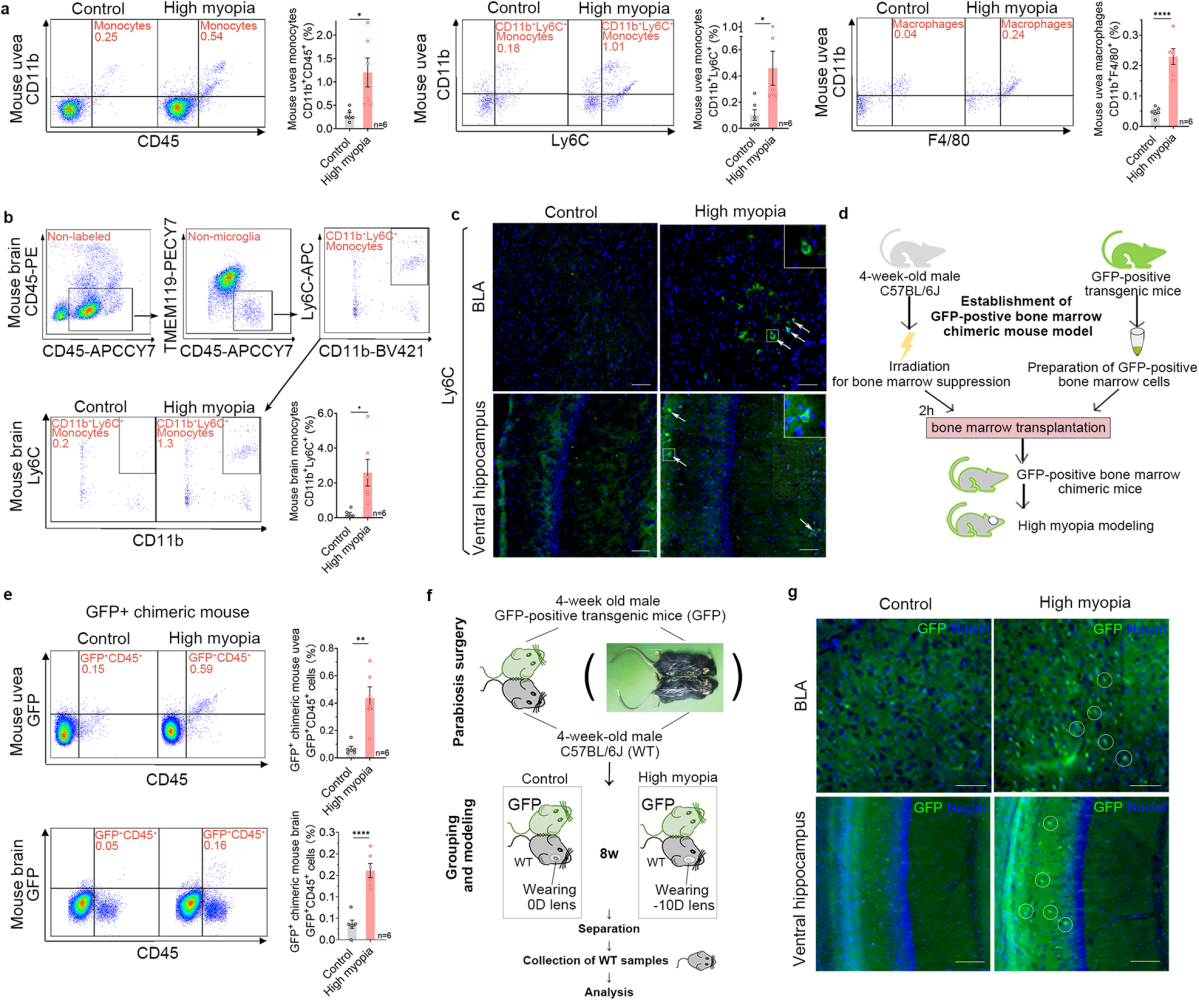
Elevated CCL2 expression increases infiltration of blood-derived monocytes/macrophages into the eyes and brains of highly myopic mice. **a** Flow-cytometric quantification showed higher ratios of monocytes (CD11b^+^/CD45^+^), CD11b^+^/Ly6C^+^ monocytes, and macrophages (CD11b^+^/F4/80^+^) in the uvea of highly myopic mice than in the controls. **b** Flow-cytometric gating strategy showed exclusion of CD45-PE labeled cells and microglia (TMEM119^+^) before quantification of infiltrated monocytes. A significantly higher ratio of CD11b^+^/Ly6C^+^ monocytes was identified in the brain of highly myopic mice than in the controls. **c** Immunofluorescent images showed increased Ly6C^+^ monocyte infiltration (arrows) into the BLA and ventral hippocampus of highly myopic mice. Enlarged images in white squares are shown on the top right. Scale bars: 100 μm. **d** Schematic representation of the establishment of a model of GFP-positive bone marrow chimeric mice. **e** Flow-cytometric quantification showed higher ratios of GFP^+^CD45^+^ cells in both the uvea and brain of GFP-positive bone marrow chimeric mice with high myopia than in the chimeric mice without high myopia. **f** Schematic representation of the establishment of the parabiotic model. **g** Immunofluorescent images showed increased GFP^+^ cell infiltration (circles) into the BLA and ventral hippocampus of parabiotic wild-type mice with high myopia than in the controls. Scale bars: 100 μm. Data are mean ± SD. *n* = 6 in each group (**a**, **b**, **e**); *n* = 3 in each group (**c**, **g**). The level of significance was detected using Student’s *t*-test. ****P < 0.0001, **P < 0.01 and *P < 0.05.

To investigate the role of CCL2 elevation in high myopia-related anxiety, we observed the infiltration of monocytes/macrophages in the eye and brain with flow cytometry. We found significantly higher ratios of monocytes (CD11b^+^/CD45^+^), CD11b^+^/Ly6C^+^ monocytes, and macrophages (CD11b^+^/F4/80^+^) in the uvea of highly myopic mice compared to the controls (1.20 ± 0.76% vs 0.29 ± 0.13% in the control, *P* = 0.0312; 0.46 ± 0.32% vs 0.10 ± 0.10% in the control, *P* = 0.0233; and 0.23 ± 0.06% vs 0.05 ± 0.02% in the control, *P* < 0.0001; Fig. 3a), suggesting increased infiltration of monocytes/macrophages in highly myopic eyes.

Meanwhile, we also identified significantly higher ratios of CD11b^+^/Ly6C^+^ monocytes in the brains of the highly myopic mice compared to the controls (2.59 ± 1.88% vs 0.23 ± 0.21% in the control, *P* = 0.0273) after exclusion of the immune cells inside blood vessels and brain microglia (Fig. 3b; Supplementary Fig. S5a), suggesting a simultaneous increase in monocyte infiltration in the brains of highly myopic mice. We further performed immunofluorescent staining of the brain and confirmed the increased Ly6C^+^ monocyte infiltration into the anxiety-related areas including the basal lateral amygdala (BLA) and ventral hippocampus in highly myopic mice (Fig. 3c). After recovery of high myopia, Ly6C^+^ monocyte infiltration was reduced in the eye but not reversed in the brain (Supplementary Fig. S3d, e).

To find the source of the infiltrated mononuclear cells in the eyes and brains of highly myopic mice, we further established a model of GFP-positive bone marrow chimeric mice, in which high myopia was induced (Fig. 3d). We found that the chimeric mice with high myopia presented significantly increased ratio of GFP^+^/CD45^+^ cells in both the uvea (0.44 ± 0.19% vs 0.07 ± 0.04% in the control, *P* = 0.0012) and the brain (0.16 ± 0.04% vs 0.04 ± 0.02% in the control, *P* < 0.0001) (Fig. 3e). And to rule out the effect of irradiation on BBB, we established a parabiotic mouse model, in which high myopia was also induced (Fig. 3f) and found increased GFP^+^ cell infiltration into the BLA and ventral hippocampus of the parabiotic wild-type mice with high myopia than in the controls (Fig. 3g). These results indicate that the infiltrated mononuclear cells in the eyes and brains of highly myopic mice were recruited from the blood.

We investigated the effect of high myopia on microglia and found no difference in the ratio of brain microglia (TMEM119^+^) between the control and highly myopic mice (Supplementary Fig. S5b). We found increased expression of Iba-1 in the brain of highly myopic mice compared to the controls (Supplementary Fig. S5c). Moreover, administration of CC chemokine receptor 2 (CCR2) inhibitor that blocked the monocyte chemotactic activity, substantially attenuated the anxiety-like behaviors in the highly myopic mice in OFT and EPM (Supplementary Fig. S5d, e), suggesting a key role of the infiltrated monocytes in the high myopia-related anxiety.

### Increased monocyte/macrophage infiltration further disrupts the BOB/BBB in highly myopic mice

**Fig. 4:**
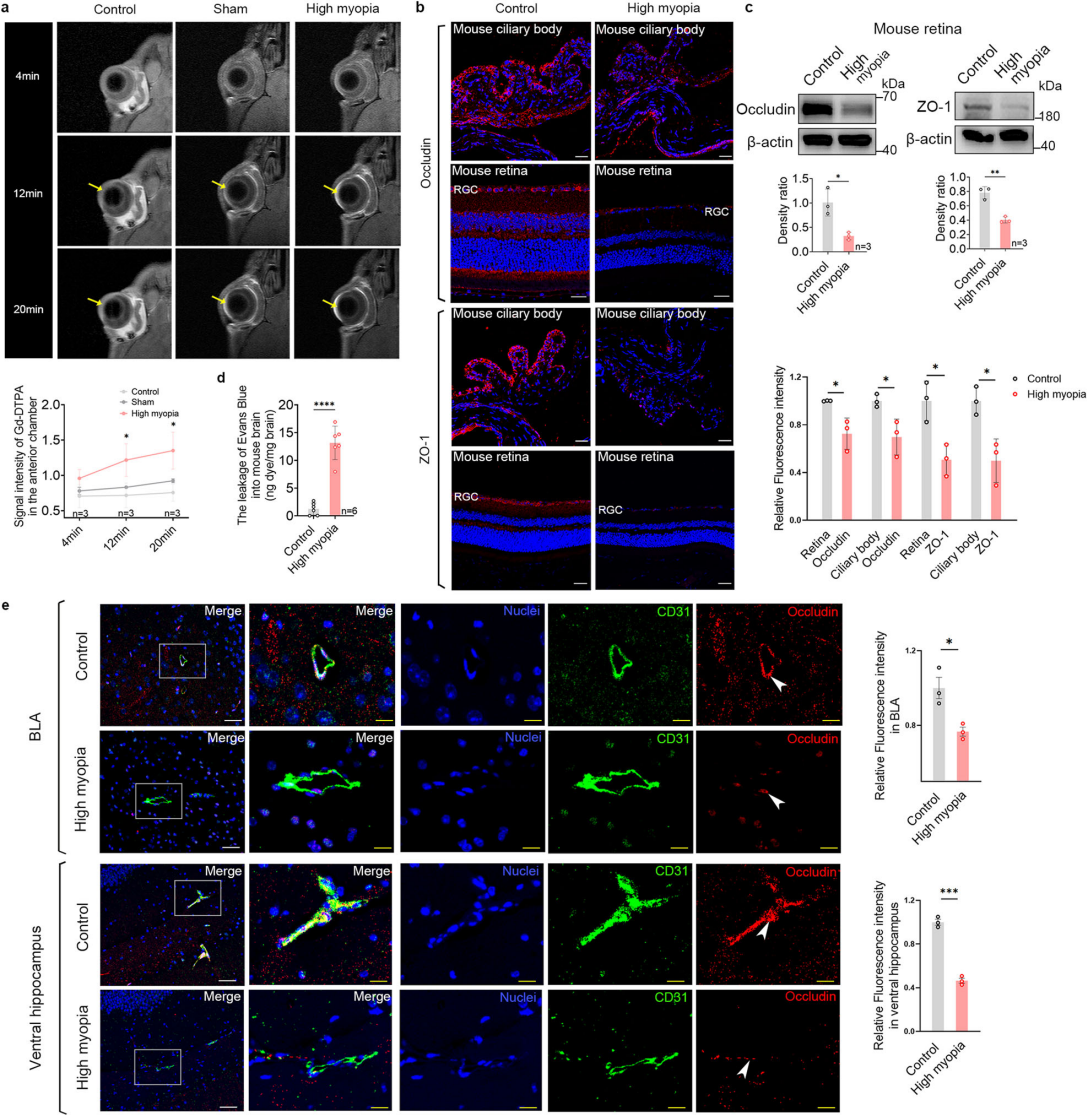
Increased monocyte/macrophage infiltration further disrupts the BOB/BBB in highly myopic mice. **a** Representative enhanced MRI images (coronal) showed faster leakage of contrast agents into the anterior chamber (yellow arrows) of highly myopic eyes than those into eyes of the control or sham eyes at both 12 min and 20 min after Gd-DTPA injection. **b** Immunofluorescent images of tight junction proteins occludin and ZO-1 in the ciliary body and retina of mice. Scale bars: 25 μm. Fluorescence intensity normalized to the averaged value of the control for quantification analyses. **c** Examination of occludin and ZO-1 expression in mouse retina with western blotting. Band density normalized to that of the loading control for statistical analysis. **d** Leakage of EB into the mouse brain (ng dye/mg brain tissue). **e** Immunofluorescent images of tight junction protein occludin (red) and vessel marker CD31 (green) in the BLA and ventral hippocampus of mice. Enlarged images of CD31-labeled vessels (square) showing downregulated occludin (arrowheads) in highly myopic mice. Scale bars: 40 μm in white and 20 μm in yellow. Fluorescence intensity normalized to the averaged value of the control for quantification analyses. Data are mean ± SD. *n* = 3 in each group (**a**–**c**, **e**); *n* = 6 in each group (**d**). Level of significance was detected with one-way repeated measures ANOVA (**a**) or Student’s *t* test (**b**–**e**). ****P < 0.0001, ***P < 0.001, **P < 0.01, *P < 0.05.

We used enhanced MRI to investigate the alteration of the BOB in mice and found that more leakage of contrast agent into the anterior chamber of the highly myopic eyes compared to that into the control and sham eyes at both 12 min and 20 min after Gd-DTPA injection (Fig. 4a), suggesting disruption of the blood–aqueous barrier (BAB) in highly myopic eyes. We also found that the tight junction proteins occludin and ZO-1 were significantly downregulated in the ciliary body and retina of highly myopic eyes, suggesting the disruption of both the BAB and blood–retina barrier (BRB; Fig. 4b, c).

Using Evans Blue (EB) staining, we identified significantly more leakage of EB into the brains of highly myopic mice than into those of the controls (Fig. 4d). We also found that the tight junction protein occludin was downregulated in the CD31-labeled vessels of BLA and ventral hippocampus in the highly myopic group (Fig. 4e), corroborating BBB disruption in highly myopic mice.

### *Ccl2* deficiency or CCL2 blockage substantially attenuates high myopia-related anxiety by reducing monocyte/macrophage infiltration and BOB/BBB disruption

**Fig. 5:**
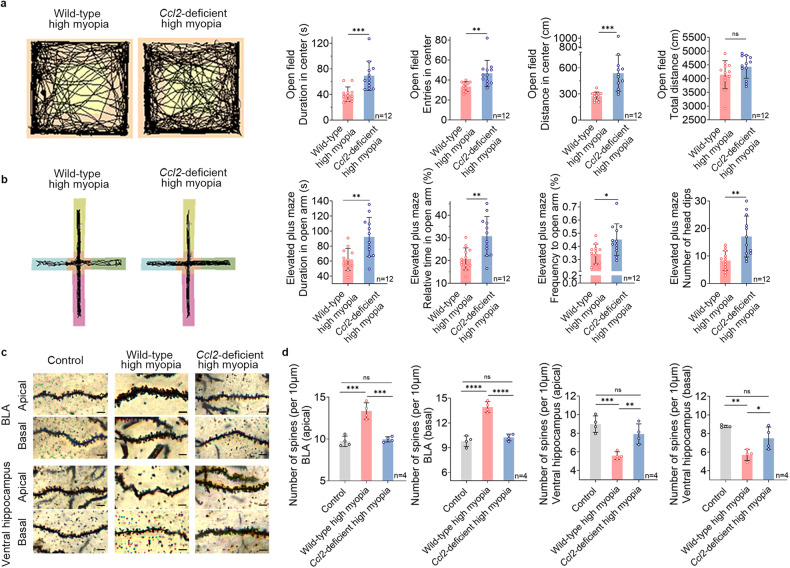
*Ccl2* deficiency substantially attenuates anxiety and reverses anxiety-related neuronal changes in highly myopic mice. **a** Representative maps of animal tracking (black lines) in the dark showed significantly reduced anxiety levels in *Ccl2*-deficient highly myopic mice than in wild-type highly myopic mice in OFT. **b** Representative maps of animal tracking (black lines) in the dark showed significantly reduced anxiety levels in *Ccl2*-deficient highly myopic mice than in wild-type highly myopic mice in the EPM test. **c** Representative Golgi images of the dendritic spines in BLA and ventral hippocampus of mice. Scale bars: 5 μm. **d** High myopia modeling increased the spine density of BLA pyramidal neurons and decreased the spine density of ventral hippocampus neurons, which were partially reversed by *Ccl2* deficiency. Data are mean ± SD. *n* = 12 in each group (**a**, **b**); *n* = 4 in each group (**c**, **d**) . The level of significance was detected with Student’s *t-*test (**a**, **b**) or one-way ANOVA (**d**). ****P < 0.0001, ***P < 0.001, **P < 0.01, *P < 0.05, ns *P* > 0.05.

To examine the role of CCL2 in high myopia-related anxiety, we used a *Ccl2*-deficient mouse model. Compared with wild-type highly myopic mice, *Ccl2*-deficient highly myopic mice displayed significantly reduced anxiety levels during OFT and EPM tests, in both dark and light conditions (Fig. 5a, b; Supplementary Fig. S6a), which was also confirmed by the Vogel test (Supplementary Fig. S6b). We then injected C57BL/6J mice with CCL2-neutralizing antibody during high myopia modeling and found that these mice also displayed substantially less anxiety in OFT and EPM tests (Supplementary Fig. S6c, d), suggesting that high myopia-related anxiety can be alleviated by either *Ccl2*-knockout or CCL2 blockage. Golgi staining of brain tissues showed that high myopia modeling increased the spine density of both apical and basal dendrites of BLA pyramidal neurons and decreased the spine density of both apical and basal dendrites of ventral hippocampus neurons compared with the controls, all of which were partially reversed by *Ccl2*-deficiency (Fig. 5c, d), corroborating the role for CCL2 in anxiety-related neuronal changes.

**Fig. 6:**
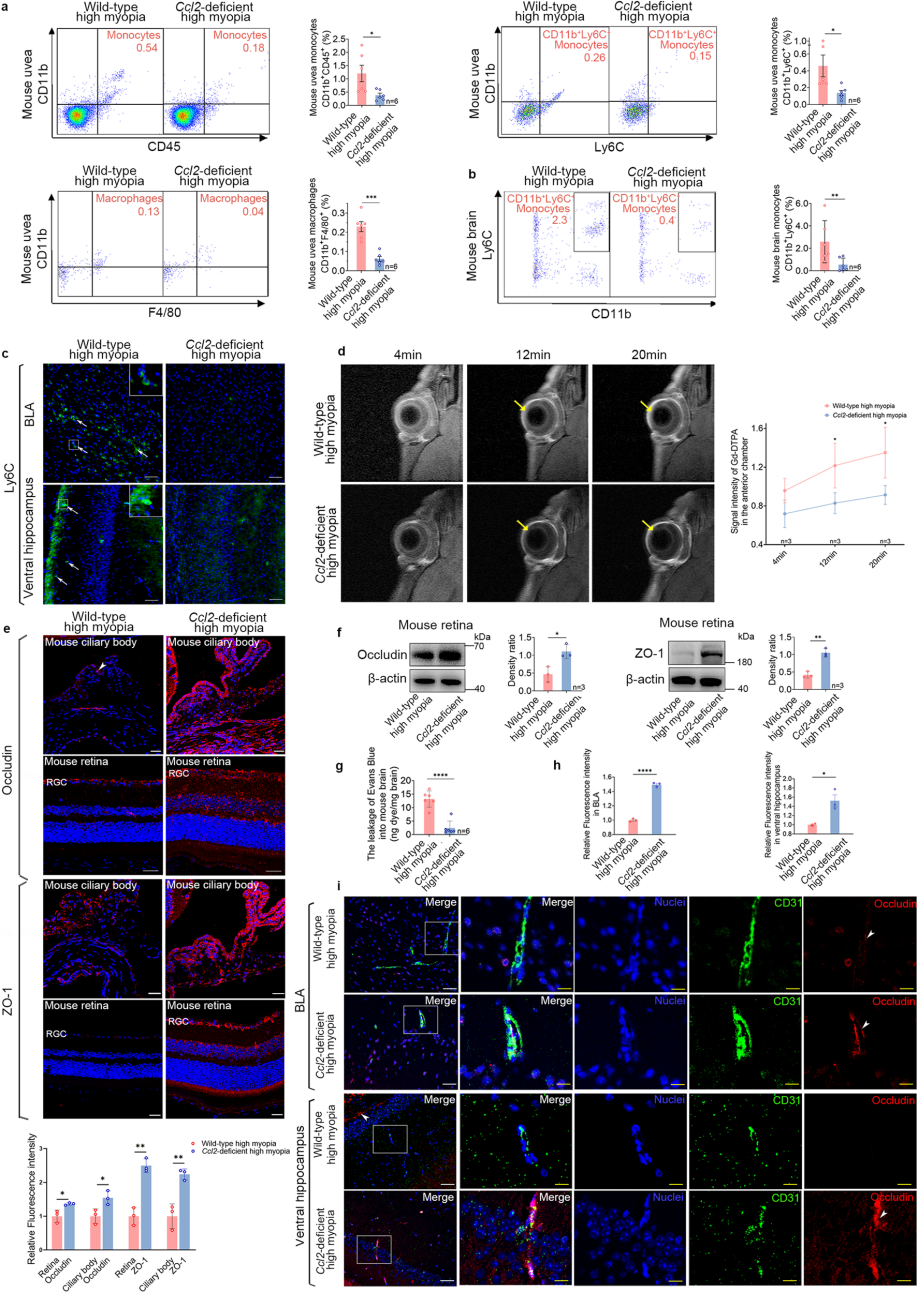
*Ccl2* deficiency reduces ocular and brain infiltration of monocytes/macrophages and BOB/BBB disruption in highly myopic mice. **a** Flow-cytometric quantification showed decreased ratios of monocytes (CD11b^+^/CD45^+^), CD11b^+^/Ly6C^+^ monocytes, and macrophages (CD11b^+^/F4/80^+^) in the uvea of *Ccl2*-deficient highly myopic mice than in those of wild-type highly myopic mice. **b** Flow-cytometric quantification showed decreased ratios of CD11b^+^/Ly6C^+^ monocytes in the brain of *Ccl2*-deficient highly myopic mice than in the controls. **c** Immunofluorescent images showed decreased infiltration of Ly6C^+^ monocytes (arrows) in the BLA and ventral hippocampus of *Ccl2*-deficient highly myopic mice. Enlarged images in white squares are shown on the top right. Scale bars: 100 μm. **d** Representative images of enhanced MRI (coronal) showed slower leakage of contrast agent into the anterior chamber (yellow arrows) of *Ccl2*-deficient highly myopic mice than into those of wild-type highly myopic mice at both 12 min and 20 min after Gd-DTPA injection. **e** Immunofluorescent images of tight junction proteins occludin and ZO-1 in the ciliary body and retina of mice. Fluorescence intensity normalized to the averaged value of the control for quantification analyses. Scale bars: 25 μm. **f** Examination of occludin and ZO-1 expression in mouse retina with western blotting. The band density was normalized to that of the loading control for statistical analysis. The lane of ZO-1 for the wild-type highly myopic group was the same as that for the highly myopic group. **g** Leakage of EB into the mouse brain. **h** Relative fluorescence intensity of occludin in the BLA and ventral hippocampus area. Fluorescence intensity was normalized to the averaged value of the wild-type for quantification analyses. **i** Immunofluorescent images of occludin and CD31 in the BLA and ventral hippocampus of mice. Enlarged images of CD31-labeled vessels (square) showed upregulated occludin (arrowheads) in the *Ccl2*-deficient highly myopic mice. Scale bars: 40 μm in white and 20 μm in yellow. Data are mean ± SD. *n* = 6 in each group (**a**, **b**, **g**); *n* = 3 in each group (**c**–**f**, **h**). The level of significance was detected with one-way repeated measures ANOVA (**d**) or Student’s *t-*test (**a**, **b**, **e**–**h**). ****P < 0.0001, ***P < 0.001, **P < 0.01, *P < 0.05.

In further mechanistic investigations, we detected significantly decreased ratios of CD11b^+^/Ly6C^hi^ monocytes (0.73 ± 0.43% vs 2.99 ± 1.85%, respectively, *P* = 0.0002) and CD11b^+^/F4/80^+^ monocytes (1.77 ± 0.98% vs 4.33 ± 2.07%, respectively, *P* = 0.0002) in the blood of *Ccl2*-deficient highly myopic mice than in wild-type highly myopic mice (Supplementary Fig. S7a, b). We also identified significantly decreased ratios of monocytes (0.38 ± 0.20% vs 1.20 ± 0.76% in the control, *P* = 0.0443), CD11b^+^Ly6C^+^ monocytes (0.14 ± 0.07% vs 0.46 ± 0.32% in the control, *P* = 0.0367), and macrophages (CD11b^+^/F4/80^+^, 0.06 ± 0.03% vs 0.23 ± 0.06% in the control, *P* = 0.0002) in the uvea of the *Ccl2*-deficient highly myopic mice (Fig. 6a), suggesting that the infiltration of monocytes/macrophages into the eye was reduced by *Ccl2* deficiency. We also detected significantly decreased ratios of CD11b^+^/Ly6C^+^ monocytes in the brain of *Ccl2*-deficient highly myopic mice (0.55 ± 0.58% vs 2.59 ± 1.88% in the control, *P* = 0.0087, Fig. 6b). Immunofluorescent staining of brain showed reduced Ly6C^+^ monocyte infiltration into the BLA and ventral hippocampus of the *Ccl2*-deficient highly myopic mice (Fig. 6c), suggesting that the infiltration of monocytes into the brain was reduced by *Ccl2* deficiency. No difference in the ratio of brain microglia (TMEM119^+^) was found between the groups (*P* = 0.2530, Supplementary Fig. S7c).

We used ocular-enhanced MRI and brain staining to examine the effects of *Ccl2* deficiency on the BOB and BBB. Enhanced MRI showed that the leakage of contrast agent into the anterior chamber was significantly slower in the *Ccl2*-deficient highly myopic mice than in the wild-type highly myopic mice at both 12 min and 20 min after Gd-DTPA injection (Fig. 6d), indicating that *Ccl2* deficiency exerts a protective effect on the BAB. The upregulation of the tight junction proteins occludin and ZO-1 in both the ciliary body and retina of the *Ccl2*-deficient highly myopic mice suggests reduced disruption of the BAB/BRB (Fig. 6e, f). Meanwhile, the leakage of EB into the brain was significantly lower in the *Ccl2*-deficient highly myopic mice than in the wild-type highly myopic mice (Fig. 6g). We also found upregulated occludin in the CD31-labeled vessels of BLA and ventral hippocampus areas in the *Ccl2*-deficient highly myopic group (Fig. 6h, i). These results together suggest less BBB disruption induced by high myopia modeling after *Ccl2* deficiency.

## Discussion

High myopia is a great public health issue due to its surging prevalence and vision-threatening complications^1–3^. The anxiety of subjects with high myopia, which can impair their mental well-being and add to the difficulties encountered during treatment, has recently become a new concern of ophthalmologists. However, the anxiety among high myopes remains poorly defined, as is the underlying mechanism. In this study, we confirmed higher risk of anxiety in highly myopic patients and mice, accompanied by higher CCL2 expression and monocyte levels in their blood. Mechanistic studies showed that highly myopic visual stimulation increased CCL2 expression in the eyes, which led to increased monocyte/macrophage infiltration in the eyes and brain, BOB/BBB disruption, and consequently more anxiety in mice. *Ccl2* deficiency or CCL2 blockage could substantially alleviate high myopia-related anxiety, probably by attenuating the infiltration of monocytes/macrophages and disruption of BOB/BBB. Together, our findings confirm the association between anxiety and high myopia for the first time, and demonstrate that CCL2-mediated inflammatory pathogenesis is the underlying mechanism of this anxiety.

We have established an association between high myopia and anxiety in this study for the first time. In clinical practice, highly myopic patients often require excessive explanations from their doctors or dwell continuously on every possible risk, making their treatment more difficult and compromising their quality of life. However, no previous investigation has examined this phenomenon comprehensively. In this study, we identified an increased risk of anxiety in highly myopic patients, who showed a greater risk of mild to moderate or severe anxiety and more psychic symptoms. This anxiety was confirmed in a mouse model of high myopia. The neuropsychiatric traits observed in highly myopic subjects are similar to those observed in patients with chronic inflammatory diseases, including rheumatoid arthritis, multiple sclerosis, and metabolic syndrome^20–22^. Therefore, we speculated that high myopia-related anxiety may also have an inflammatory pathogenesis, which had not been investigated previously.

Elevated ocular CCL2, driven by highly myopic visual stimulation, may be the initiator of high myopia-related anxiety. Under chronic stimulation, chemokine production may be upregulated in the neuroretina, further activating a kind of para-inflammation^23^. Therefore, prolonged highly myopic visual stimulation induced by −10 D lenses may also induce chemokine production in the neuroretina, as indicated by the increased CCL2 expression in the PRC and RGC layers of the highly myopic mice in the present study. Upregulated CCL2 in the PRC layer implies that myopic visual simulation may lead to the abnormal conversion of the light signal to an electrical signal and local inflammation^24^. Increased CCL2 expression in the RGC layer suggests that sustained myopic visual simulation may further cause abnormal delivery of vision-related signals to higher visual centers by the RGC and local inflammation^25,26^. Besides, mechanical force stimulation generated by the AL change of the eyes may be a secondary factor affecting inflammation. Studies have shown that AL change may be a result of myopic visual stimulation and myopia-induced inflammation^27–29^. Whether the mechanical force stimulation further affects inflammation needs to be explored in future study. CCL2 expression and monocyte infiltration in the eye were reduced by recovery of high myopia, suggesting that elevated CCL2 may be a key mediator between myopic visual stimulation and the subsequent myopia-induced inflammation.

The elevation of ocular CCL2 is followed by an increase in CCL2 and monocyte levels in the blood of highly myopic subjects. Our data show that high myopes with anxiety presented with even higher levels of blood CCL2 than those with no anxiety, and the positive association between the anxiety scores and blood CCL2 levels is similar to the findings for post-traumatic stress disorder and generalized anxiety disorder^30,31^. We also found an increased level of circulating monocytes and CD14^+^CD68^+^ monocytes that has the potential to be activated into inflammatory macrophages in highly myopic subjects^32,33^. As in highly myopic mice, we also identified a significant increase in an inflammatory monocyte subset (Ly6C^hi^) that is associated with many chronic diseases, including diabetes and atherosclerosis^34,35^. Together with the elevated inflammatory cytokines identified in the blood of highly myopic subjects, this suggests a CCL2-mediated inflammatory pathogenesis for high myopia-related anxiety.

The infiltration of blood-derived monocytes/macrophages into the eyes and brains of highly myopic subjects increased significantly as a consequence of ocular CCL2 elevation. In the highly myopic mouse model, we detected a significant increase of Ly6C^+^ monocytes in the eyes and anxiety-related brain areas including BLA and ventral hippocampus, which could be attenuated by *Ccl2* deficiency. This monocyte subset can exit the bloodstream, patrol the extravascular tissues, and differentiate into macrophages during inflammation, and their recruitment usually depends on the CCL2 pathway^19,36^. Using a GFP-positive bone marrow chimeric mouse model, in which the peripheral blood was reconstituted with the donated GFP-positive bone-marrow-derived cells and which is very useful in tracing the recruitment of bone-marrow-derived cells from blood^37,38^, we verified that the infiltrated mononuclear cells in the eyes and brains of highly myopic mice originated from the blood. Using a parabiotic mouse model, we verified the infiltration of blood-derived GFP^+^ cells into BLA and the ventral hippocampus. In the eye, inflammatory monocyte/macrophage infiltration causes tissue injury in maculopathy, glaucoma, and retinitis pigmentosa, and in the brain, it generates anxiety-like behavior after blast injury and social stress^39–44^. Therefore, ocular and brain infiltration of monocyte/macrophage may induce local tissue injury and cause high myopia-related anxiety.

Increased monocyte/macrophage infiltration further renders the disruption of BOB/BBB. The BOB mainly consists of the tight junctions in the nonpigmented epithelium of the ciliary body (BAB) and the retinal pigment epithelium and vessels (BRB), whereas the BBB is mainly created by tight junctions of the vascular endothelium and the basement membrane^13,45,46^. Their breakdown occurs during ocular inflammation or neuronal pathologies, such as psychiatric disorders, epilepsy, and stroke^14,15,47–49^. In this study, we confirmed the reduced function of the BOB/BBB after the induction of high myopia, which was reversed by *Ccl2* deficiency, suggesting a role for CCL2 in the high myopia-related disruption of BOB/BBB.

**Fig. 7:**
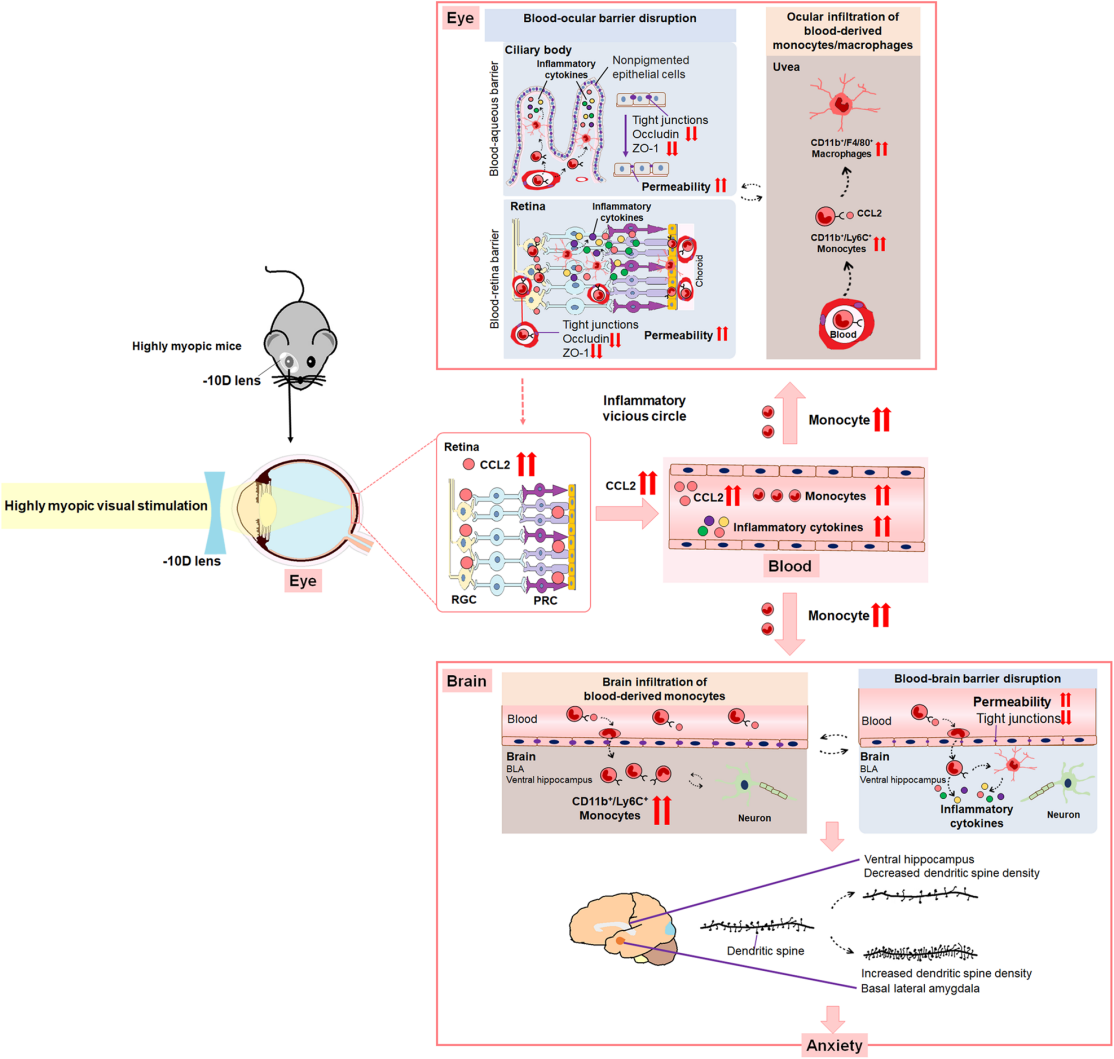
Schematic illustration of CCL2-mediated inflammatory pathogenesis that underlies high myopia-related anxiety. Highly myopic visual stimulation upregulates ocular CCL2 expression and then increases blood CCL2 and monocyte levels, which increases the ocular and brain infiltration of monocytes/macrophages. Ocular infiltration further exacerbates BOB disruption and CCL2 release into the circulation, aggravating an inflammatory vicious circle. Brain infiltration further disrupts BBB, exacerbates local inflammation and alters dendritic spines, eventually leading to anxiety.

The disruption of the immune barriers further aggravates local inflammation, leading to a vicious circle. Interestingly, the highly myopic mice showed increased monocyte infiltration, BBB disruption, and altered dendritic spine density in BLA and ventral hippocampus, which are closely related to anxiety^50–52^, implying a potential relationship among inflammation, BBB disruption and high myopia-related anxiety. The cytokines secreted by infiltrated leukocytes can cause BBB breakdown^53–55^, which has been seen in anxiety-related diseases such as chronic stress, neuropsychiatric lupus, and bipolar disorder^15,56,57^. In lipopolysaccharide-induced inflammation, a long-term immune response is activated in the peripheral blood and brain, leading to disruption of the BBB, neuronal dysregulation, and eventually to anxiety in mice^58,59^. Therefore, BBB disruption in highly myopic subjects may also be closely associated with anxiety. We propose the potential mechanism underlying high myopia-related anxiety as follows (Fig. 7). Highly myopic visual stimulation upregulates CCL2 expression in the eye, and then increases CCL2 and monocyte levels in the circulation, which further increases the ocular and brain infiltration of monocytes/macrophages. Ocular infiltration further exacerbates BOB disruption and CCL2 release into the circulation, aggravating an inflammatory vicious circle. Brain infiltration further renders BBB disruption, local inflammatory response and dendritic spine alterations, eventually leading to anxiety. Notably, activation of microglia may probably participate in remodeling synapses^60,61^. Infiltrating macrophages can affect microglia-mediated phagocytosis^61^. Besides phagocytosis, microglia can also modulate synapses via secreting immune signals which have varied effects on the inhibitory and excitatory synapses^62^. Future research will investigate how the microglia is activated and regulates neuronal function in the context of high myopia-related anxiety.

Some issues of the study need to be addressed. Highly myopic patients tend to have higher risk of anxiety than the normal population, so we mainly focused on those with anxiety in the study. Part of the highly myopic patients did not show significant elevation of blood CCL2 and anxiety. One possibility is that adaptation happens over time so that eventually myopia-induced inflammation calms down and the anxiety is gone. Future longitudinal study will be conducted. Low or moderate myopia was not included in the study. Studies showed that low or moderate myopia might have little influence on anxiety^6,63^. Perhaps patients with low to moderate myopia merely show short-sightedness of the eye but do not have significant pathological or systematic damage^64,65^, making them less anxious. Further research focusing on this population may be needed. Moreover, like many neurodegenerative pathologies^66–68^, we found during the development of high myopia, chronic inflammatory infiltration in the brain and blood-brain barrier disruption that had already occurred might persist even after the recovery of high myopia, though the ocular inflammation could be attenuated. Therefore, finding the interventional target for brain infiltration and damage is necessary. Yet, this finding may need to be further verified with a longer recovery period in future research.

In summary, our study establishes the association between high myopia and anxiety for the first time and highlights the CCL2-mediated inflammatory pathogenesis as its underlying mechanism. We provide a therapeutic target for the management of high myopia-related anxiety and establish a potential connection between visual stimulation and psychiatric disorder.

## Materials and methods

### Ethical approval

This study was reviewed and approved by the Ethical Committee of the Eye & Ear, Nose, and Throat Hospital of Fudan University, Shanghai, China (no: 2020068). All experiments were performed in accordance with the Declaration of Helsinki and the relevant guidelines and regulations. Written informed consent was obtained from each subject. Animal experiments conformed to the ARVO Statement for the use of animals in research.

### Patients

In this study, highly myopic patients were defined as those with AL ≥ 26 mm in both eyes, and the control group was defined as emmetropic patients with AL between 22 mm and 24.5 mm in both eyes.

### Patients for anxiety evaluation and blood sample collection

To compare the anxiety levels between the highly myopic and control groups, patients who visited our outpatient clinic were interviewed. The exclusion criteria included uveitis, glaucoma, trauma, previous psychological or cognitive disorder, use of any immunomodulating medication, any inflammatory event within the 3 months preceding participation, and other systemic diseases, such as diabetes or tumor.

Anxiety was evaluated by the instructed physician using the 14-item HAMA questionnaire. Each item had a score of 0–4, representing the absence or severity of the symptom. The total score, ranging from 0 to 56, was categorized as follows: no anxiety (0–6), mild to moderate anxiety (7–13), severe anxiety (≥ 14). The 14 items were divided into two subscales: psychic anxiety (anxious mood, tension, fears, depressed mood, behavior at the interview, insomnia, and cognition) and somatic anxiety. Ultimately, questionnaires from 210 patients were collected.

To evaluate CCL2 and monocyte levels, blood samples were also collected from these patients. Blood was collected in 5 mL EDTA-treated tubes between 9 a.m. and 11 a.m. For the cytokine array, plasma was obtained from 2 mL of fresh blood by centrifugation at 1500× *g* for 10 min at 4 °C and was stored in aliquots at −80 °C. For flow cytometry, peripheral blood mononuclear cells (PBMCs) were isolated from 2 mL of fresh blood with a Ficoll gradient (GE Healthcare, cat. no. 17-1140-02) before they were stained for flow cytometry. The plasma (100 μL) or PBMCs from one individual were used as one sample for analysis with the cytokine array or flow cytometry.

### Animals

Mice were bred and housed in clear open cages (5 per cage) at Shanghai SLAC Laboratory, Shanghai, China under a 12 h light/dark cycle at 21 °C and 40%–60% humidity, with access to water and standard chow diet ad libitum.

### Defocus-induced highly myopic mouse model

Four-week-old male C57BL/6J mice were used to establish the defocus-induced highly myopic model by positioning a −10 D lens on the periorbital skin of the right eye. At the beginning of the study, the refraction in both eyes was measured during wakefulness with an automated eccentric infrared photorefractor (Steinbeis Transfer Center, Stuttgart, Germany), and mice with binocular refraction difference >1 D were excluded. The lenses were checked every day and repositioned if necessary. After 4 weeks, refraction was measured with the same method, and only mice with a myopic shift in refraction in the right eye that was at least 6 D larger than that in the left eye were regarded as a successful model of high myopia. Mice wearing plano lenses of 0 D on the right eye were used as the sham-treated control group to eliminate the effect of wearing any lens on the mice’s behavior.

### GFP-positive bone marrow chimeric mice

EGFP-positive transgenic mice (C57BL/6JSmoc-Gt(ROSA)26Sor^em2(CAG-EGFP-WPRE-polyA)Smoc^, GFP-positive transgenic mice for short) were provided by the Immunology Laboratory of the Second Military Medical University, Shanghai, China. The bone marrow of the GFP-positive transgenic mice was extracted by preparing and flushing the bones with Dulbecco’s modified Eagle’s medium (DMEM; Sigma-Aldrich, USA) containing 10% fetal bovine serum (FBS; Gibco, USA, cat. no. 10099141). The GFP-positive bone-marrow cells were washed with phosphate-buffered saline (PBS), treated with red blood cell lysing buffer (BD Biosciences, cat. no. 555899), and filtered through 0.22-μm mesh. The concentration of GFP-positive bone-marrow cells in suspension was adjusted to 1 × 10^6^ cells/100 μL before transplantation. Four-week-old male C57BL/6J mice were irradiated with 6–8 Gy to inhibit hematopoiesis in their own bone marrow, and after 2 h, they were injected intraperitoneally with the GFP-positive cell suspension (150 μL per mouse). The GFP-positive chimeric mice were then subjected to high myopia modeling.

### Parabiotic mouse model

Four-week-old male C57BL/6J mice and EGFP-positive transgenic mice (C57BL/6JSmoc-Gt(ROSA)26Sor^em2(CAG-EGFP-WPRE-polyA)Smoc^, GFP-positive transgenic mice for short) were surgically conjoined according to previous studies^69^. In a parabiotic pair, the wild-type mouse was then subjected to high myopia modeling. After 8 w, parabiotic pairs were disjoined.

### Mice treated with CCR2 inhibitor

Four-week-old male C57BL/6J mice were orally administrated with a CCR2 inhibitor (RS504393, 2 mg/kg; MedChemExpress, cat. no. HY-15418) every day during the period of high myopia modeling to block the monocyte chemotactic activity. Mice given the same dose of saline were used as the control.

### *Ccl2*-deficient highly myopic mice

*Ccl2*-deficient mice (*Ccl2-KO*; B6.129S4-Ccl2^tm1Rol^/J, IMSR_JAX:004434) were provided by the Jackson Laboratory (https://www.jax.org/strain/004434). A targeting vector containing neomycin-resistance and herpes simplex virus thymidine kinase genes was used to disrupt exon 2 of the mouse *Ccl2* gene and insert an in-frame stop codon into exon 1. The construct was electroporated into 129S4/SvJae-derived J1 embryonic stem cells, and the correctly targeted cells were further injected into C57BL/6 blastocysts. Obtained chimeric animals were crossed to C57BL/6 mice, and then backcrossed to C57BL/6 for ten generations. The mutant mouse is a useful model for studying leukocyte trafficking because no *Ccl2* gene product is detectable in the homozygous mouse. Four-week-old male *Ccl2*-deficient mice were subjected to high myopia modeling, and wild-type highly myopic mice were used as the control.

### Mice treated with CCL2-neutralizing antibodies

Four-week-old male C57BL/6J mice were injected intraperitoneally with a rabbit anti-mouse CCL2 polyclonal antibody (50 μg per mouse, 0.5 mg/mL; Abmart Corporation, Shanghai, China) twice a week during the period of high myopia modeling to neutralize the CCL2 protein. Mice injected with the same volume of saline were used as the control.

### Acquisition, preservation, and usage of mouse samples

Mice were anesthetized with an intraperitoneal injection of 0.1 mL of 10% chloral hydrate. Their eyeballs were enucleated and fixed in 4% paraformaldehyde for sectioning. To isolate the retina and uvea, dissection was performed with fine forceps and ophthalmic scissors immediately after the enucleation of the eyeball. The retinas were stored at −80 °C after isolation until further analysis. Fresh uveas were cut into pieces, digested in digestion medium (DMEM, 10% FBS and 0.02 mg/mL collagenase type IV (Sigma-Aldrich, cat. no. C5138)) at 37 °C for 2 h with shaking at 70 rpm. They were then washed with PBS and filtered through a 70-μm mesh nylon cell strainer before they were stained for flow cytometry.

Blood from retro-orbital bleeds was collected into 1.5 mL EDTA-coated centrifuge tubes. For the cytokine array analysis, plasma was separated from the blood by centrifugation at 3000× *g* for 20 min at 4 °C and stored in aliquots at −80 °C. For flow cytometry, fresh blood was prepared for staining after red blood cell lysis.

Brain tissue was isolated after the transcardial perfusion of mice with PBS. For flow cytometry, the fresh brain was minced into small pieces, incubated in a digestion medium, and passed through a 70-μm mesh nylon cell strainer. Then brain mononuclear cells were isolated with Percoll density gradient centrifugation (GE Healthcare, cat. no. 17-0891-02). For immunofluorescence, the brain was fixed in 4% paraformaldehyde.

The retina from one eye, the brain, the plasma (50 μL), and the blood from one mouse were used as single samples. Mouse uveal samples (4–6 pieces) were pooled into one sample for flow cytometry because each piece contained a limited number of cells.

### Mouse ocular MRI

Mouse ocular MRI was performed with a high-resolution 7.0 Tesla MRI system (BioSpec 70/20 USR, Brucker). AL was measured with ImageJ software (https://imagej.nih.gov/ij/). Enhanced MRI with gadolinium-diethylenetriaminepentaacetic acid (Gd-DTPA; Magnevist, Bayer) was used to evaluate the BAB. High-resolution T1-weighted MRI images were recorded before and 4 min, 12 min, and 20 min after the intraperitoneal administration of 10% Gd-DTPA (0.5 mL per mouse). To analyze the leakage of contrast agent, the signal intensity of the anterior chamber on the MRI images was measured with ImageJ software and normalized to that before injection for statistical analysis.

### Behavioral analyses

The OFT and EPM were used to evaluate the anxiety-like behaviors of the mice in both dark and light conditions. In the OFT, the mice were placed individually in the corner of the Plexiglas® test apparatus (40 × 40 × 25 cm^3^) and their activity was recorded for 10 min. The time spent in the center (20 × 20 cm^2^), entries to the center, distance traveled in the center and total distance in the open field were measured with an automated tracking system (EthoVision XT 12, Noldus Technologies, USA). The EPM consisted four cross-shaped arms (36 cm long and 6 cm wide) made of opaque gray polypropylene and elevated 75 cm above the floor. The two closed arms were enclosed with 19 cm high opaque walls while the other two open arms were surrounded with 0.3 cm edges. The mice were placed in the center facing the open arm, and their activity was recorded for 5 min. The relative time (in percentage) spent in the open arms and the frequency of entry into the open arms (open-arm entries/total arm entries) were analyzed with the automated tracking system. The number of head dips was also recorded. OFT and EPM were performed at an interval of 24 h to allow the mice sufficient relaxation in the home cage between tests. Mice did not wear lenses during tests. To mimic human conditions wearing lenses, tests were also performed during which highly myopic mice wearing a +10 D lens on top of –10 D lens for myopic correction. In a rescue experiment, the –10 D lens was removed from the highly myopic mice after the successful modeling, and after one week, recovery of their refraction was ensured and evaluations were performed on them. To rule out the effect of myopia-induced vision impairment on behavioral assessment, Vogel test was conducted based on the procedure described by Vogel et al. ^70^ using an anxiety monitoring system “SuperVogel” software (XINRUN Ltd, Shanghai, China).

### Inflammatory cytokine array

The inflammatory cytokines in human blood were evaluated with the Bio-Plex Pro Human Cytokine Screening Panel (Bio-Rad Laboratories, USA, cat. no. 12007283) and analyzed with BioPlex Manager 6.0 software (Bio-Rad Laboratories). The inflammatory cytokines in mouse blood were assessed with the MSD U-PLEX Proinflammatory Biomarker Kit (MSD, USA, cat. no. K15069L-1) and read with a MESO QuickPlex SQ 120 electrochemiluminescence reader (Meso Scale Diagnostics).

### Flow cytometry

Human or mouse cells were incubated with Hu Fc Block Pure Fc1.3216 (BD Biosciences, cat. no. 564219) or rat anti-mouse CD16/CD32 (BD Biosciences, cat. no. 553142), respectively, to block the Fc receptors. The cells were then stained with antibodies in a staining buffer (BD Biosciences, cat. no. 554656). The following antibodies were used to label human cells: anti-CD45 (Biolegend, cat. no. 304014, 1:100), anti-CD14 (BD Biosciences, cat. no. 563079, 1:100). Cells were fixed, permeabilized with the BD Cytofix/Cytoperm Fixation/Permeabilization Solution Kit (BD Biosciences, cat. no. 51-2090KZ) and stained with anti-CD68 antibody (Biolegend, cat. no. 333813, 1:100).

For mouse blood and uvea, cells were labeled using anti-CD45 (BD Biosciences, cat. no. 550994, 1:200), anti-CD11b (BD Biosciences, cat. no. 552850, 1:200), anti-Ly6C (BD Biosciences, cat. no. 560592, 1:200), and anti-F4/80 (BD Biosciences, cat. no. 565411, 1:200). For mouse brain, to label the immune cells inside blood vessels, a PE-conjugated anti-CD45 antibody (Biolegend, cat. no. 103106) was intravenously injected to the mice 2 min before sample collection. Brain cells were then labeled using anti-CD45 (BD Pharmingen, cat. no. 557659, 1:200), anti-CD11b (BD Pharmingen, cat. no. 562605, 1:200), anti-Ly6C (BD Pharmingen, cat. no. 560595, 1:200), and anti-TMEM119 (Thermo, cat. no. 25-6119-82, 1:200). Flow cytometry on PBMCs was conducted to verify that immune cells inside blood vessels were successfully labeled with CD45-PE.

The fluorescence-activated cell sorting data were acquired with a BD FACSCanto II flow cytometer (BD Biosciences) and analyzed with FlowJo (V10, Tree Star, Inc).

### EB staining

After anesthetization, the mice were injected intraperitoneally with 2% EB (4 mL/kg). After 24 h, the mice were perfused transcardially with saline until the fluid from the right atrium was colorless. The brain was dissected, weighed, homogenized with a tissue grinder containing 500 μL of 50% trichloroacetic acid, and incubated at 4 °C overnight. The homogenate was then centrifuged at 10,000× *g* for 20 min, and the supernatant was diluted appropriately before being added to the wells of a 96-well plate. Fluorescence intensity was measured at 620/680 nm. Absorbance values were converted to ng of dye using a standard curve of EB. The results were expressed as ng dye/mg brain tissue.

### Golgi staining

Mouse brains were fixed (Servicebio, cat. No. G1101) for > 48 h and cut into blocks with a thickness of 2–3 mm. The tissues were gently rinsed, placed in Golgi-Cox staining solution (Servicebio, cat. no. G1069) in the dark for 14 d, immersed in 80% glacial acetic acid overnight, and then transferred to 30% sucrose solution. Brain coronal sections (100 μm thick) were prepared with the cryostat (Thermo CRYOSTAR NX50) and mounted on microscope slides. The dried sections were treated with concentrated ammonia and observed using a microscope equipped with Slide Converter (3DHISTECH, Pannoramic 250). Brain areas related to anxiety including BLA and ventral hippocampus were selected for analysis. Well-isolated pyramidal neurons with intact dendritic branches were selected from three different visual fields in either hemisphere. The spine density of dendrites were measured separately for apical and basal parts with Fiji software.

### Immunofluorescence and western blotting

Tissue sections were fixed in 4% paraformaldehyde, permeabilized with PBS containing 0.5% Triton X-100, and blocked with 3% bovine serum albumin. After fluorescently probed with antibodies against CCL2 (Abcam, cat. no. ab25124, 1:100), Ly6C (Santa Cruz, cat. no. sc271811, 1:50), Iba-1 (Abcam, cat. no. ab178846, 1:1000), occludin (Proteintech, cat. no. 27260-1-AP, 1:500), ZO-1 (Invitrogen, cat. no. PA5-85256, 1:100) and CD31 (Santa Cruz, cat. no. sc18916, 1:50) overnight at 4 °C, the sections were incubated with a secondary antibody (Invitrogen, cat. no. A-11012, 1:1000) before examination with confocal microscopy (TCS SP5, Leica Microsystems, Germany). Thickness of sections for immunostaining in the eye and brain was 10 μm and 70 μm, respectively.

For western blotting, protein extracts obtained with RIPA lysis buffer were separated by SDS-PAGE and transferred to polyvinylidene difluoride membranes. After blocking, the membranes were probed with primary antibodies against CCL2 (Abcam, cat. no. ab25124, 1:1000), occludin (Proteintech, cat. no. 27260-1-AP, 1:1000), ZO-1 (Invitrogen, cat. no. PA5-85256, 1:1000), and β-actin (Sigma Chemical, cat. no. A5441, 1:5000) and then with a secondary antibody (CST, cat. no. 7076, 7074, 1:1000). The bands were visualized with enhanced chemiluminescent reagent (Beyotime, cat. no. P0018FS). Band intensities were analyzed with ImageJ software and normalized against the corresponding β-actin band.

### Statistical analysis

The normality of distributions was assessed with the Kolmogorov–Smirnov test. Continuous variables were compared between two groups with a two-sided Student’s *t*-test or the Mann–Whitney *U* test. One-way analysis of variance (ANOVA) with Tukey’s post hoc test was used for comparisons of multiple groups. One-way repeated measures ANOVA was used for comparisons among multiple time points. Categorical variables were compared with a χ^2^ test. Pearson’s correlation analysis was used to assess the correlations between variables. A *P* value of < 0.05 was considered statistically significant. All analyses were performed with Prism 8.0 (GraphPad Software, Inc., USA).

### Supplementary information

Supplementary Information

## Data Availability

The datasets generated or analyzed during the current study are available from the corresponding author upon reasonable request. Source data are provided with this manuscript.
