# Supplementary material for: CCL2-mediated inflammatory pathogenesis underlies high myopia-related anxiety
Source: Cell Discov. 2023 Sep 12;9:94. doi: 10.1038/s41421-023-00588-2 (PMC10497683; doi:10.1038/s41421-023-00588-2)
Supplement: Supplementary file 1 — Supplementary Information [file 41421_2023_588_MOESM1_ESM.pdf]

Supplementary Information for

CCL2-mediated inflammatory pathogenesis underlies high myopia-related anxiety

Xiangjia Zhu,[1,2,3,4†*] Jiaqi Meng,[1,2,3†] Chaofeng Han,[5*] Qingfeng Wu,[6] Yu Du,[1,2,3] Jiao Qi,[1,2,3] Ling Wei,[1,2,3] Hao Li,[1,2,3] Wenwen He,[1,2,3] Keke Zhang,[1,2,3] and Yi Lu[1,2,3*]

[1]Eye Institute and Department of Ophthalmology, Eye & ENT Hospital, Fudan University, Shanghai 200031, China

[2]NHC Key Laboratory of Myopia (Fudan University); Key Laboratory of Myopia, Chinese Academy of Medical Sciences, Shanghai 200031, China

[3]Shanghai Key Laboratory of Visual Impairment and Restoration, Shanghai 200031, China

[4]State Key Laboratory of Medical Neurobiology, Fudan University, Shanghai 200032, China

[5]Department of Histoembryology and Shanghai Key Laboratory of Cell Engineering, Naval Medical University, Shanghai 200433, China.

[6]State Key Laboratory of Molecular Development Biology, Institute of Genetics and Developmental Biology, Chinese Academy of Sciences, Beijing 100101, China; University of Chinese Academy of Sciences, Beijing 100101, China; CAS Center for Excellence in Brain Science and Intelligence Technology, Shanghai 200031, China; Chinese Institute for Brain Research, Beijing 102206, China.

[†]These authors contributed equally to this work.

This file includes:

Supplementary Figures S1 to S7

Supplementary Table S1

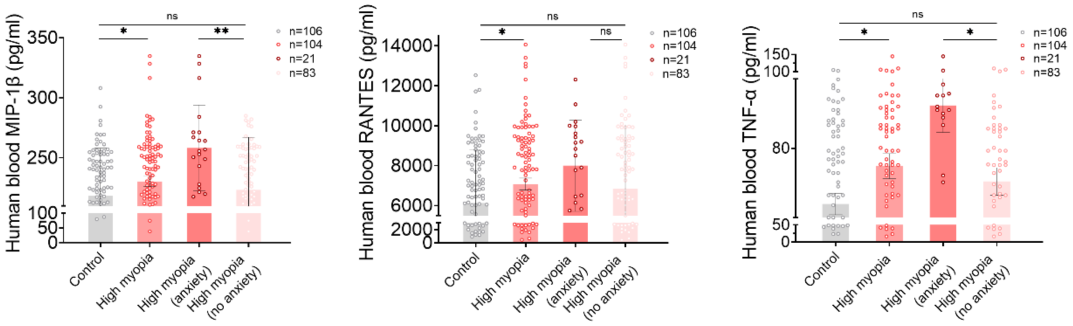

Human blood MIP-1β (pg/ml)
350
300
250
100
50
0
ns
*
**
n=106
n=104
n=21
n=83
Control
High myopia
High myopia (anxiety)
High myopia (no anxiety)
Human blood RANTES (pg/ml)
14000
12000
10000
8000
6000
2000
0
ns
*
ns
n=106
n=104
n=21
n=83
Control
High myopia
High myopia (anxiety)
High myopia (no anxiety)
Human blood TNF-α (pg/ml)
150
100
80
50
0
ns
*
*
n=106
n=104
n=21
n=83
Control
High myopia
High myopia (anxiety)
High myopia (no anxiety)

Supplementary Fig. S1. Highly myopic patients exhibit higher level of inflammatory cytokines in the blood. Measurement of MIP-1β, RANTES, and TNF-α levels in human blood with a cytokine array (n = 106 in control group, n = 104 in highly myopic group, n = 21 in highly myopic group with anxiety [total anxiety score ≥ 7], and n = 83 in highly myopic group with no anxiety). Data are means ± SD. Level of significance was detected with Student's *t* test. $^{**}P < 0.01$, $^{*}P < 0.05$, and *ns* $P > 0.05$.

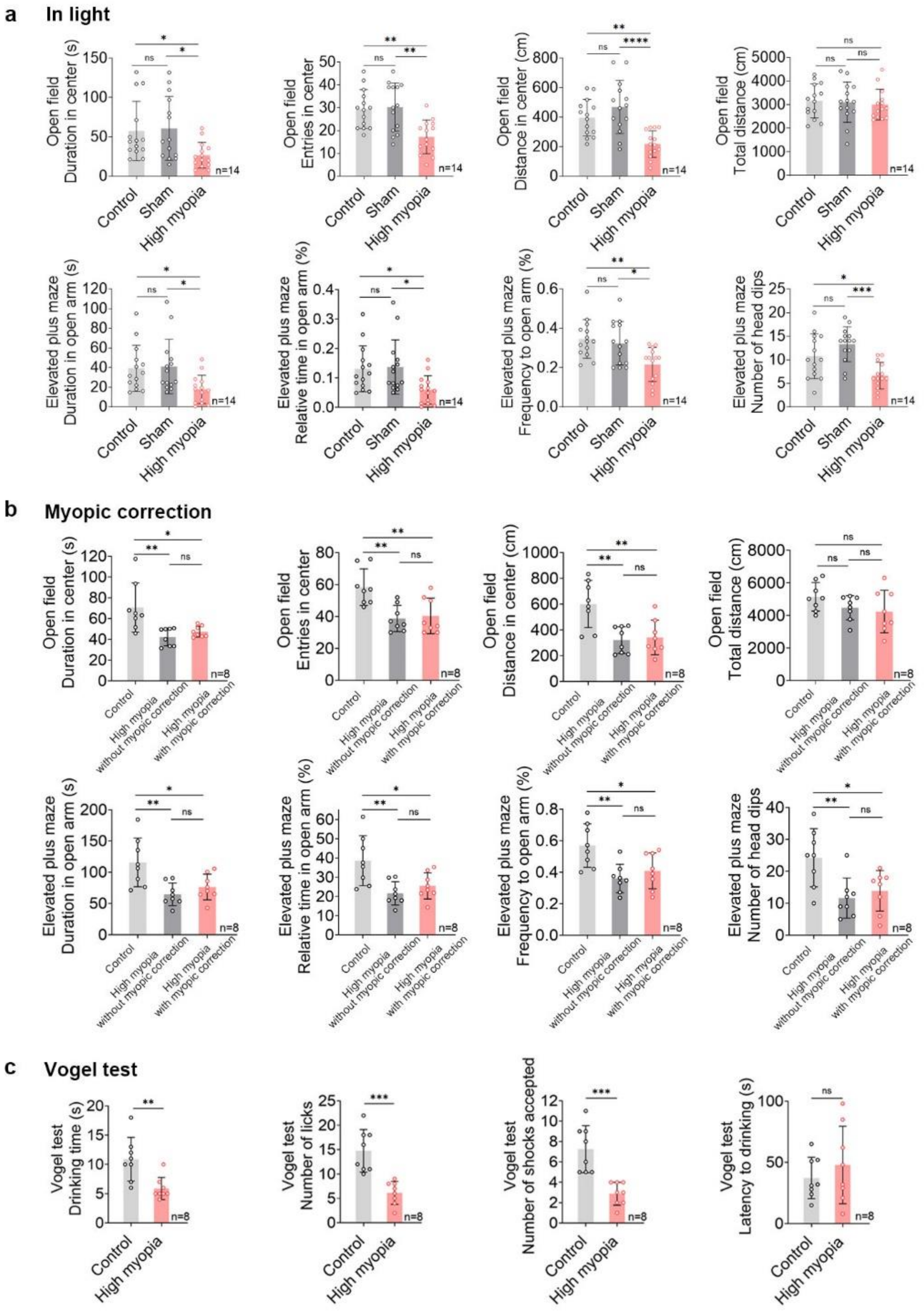
a
In light
Open field
Duration in center (s)
Open field
Entries in center
Open field
Distance in center (cm)
Open field
Total distance (cm)
Elevated plus maze
Duration in open arm (s)
Elevated plus maze
Relative time in open arm (%)
Elevated plus maze
Frequency to open arm (%)
Elevated plus maze
Number of head dips
Control
Sham
High myopia
n=14
b
Myopic correction
Control
High myopia
without myopic correction
High myopia
with myopic correction
n=8
c
Vogel test
Vogel test
Drinking time (s)
Vogel test
Number of licks
Vogel test
Number of shocks accepted
Vogel test
Latency to drinking (s)
Control
High myopia
n=8

Supplementary Fig. S2. Increased anxiety-like behaviors in highly myopic mouse model are confirmed under different conditions. (**a**) In light, the highly myopic mice exhibited increased anxiety-like behaviors than the controls, manifested as shorter duration, fewer entries, and shorter distance traveled in the center (yellow square) of the open field as well as shorter duration, fewer entries, and fewer head dips in the open arms (pink and yellow area) of the elevated plus maze. (**b**) Both open field and elevated plus maze tests showed that myopic correction during tests could not reduce the anxiety-like behaviors of highly myopic mice. (**c**) Vogel tests showed increased anxiety-like behaviors in the mouse model of high myopia, manifested as shorter drinking time, fewer licks, and fewer shocks accepted, while no difference in latency to drinking was identified between groups. (**a**) $n = 14$ in each group. (**b-c**) $n = 8$ in each group. Data are means ± SD. Levels of significance were detected with (**a, b**) one-way ANOVA or (**c**) Student's *t* test. ****$P < 0.0001$, ***$P < 0.001$, **$P < 0.01$, *$P < 0.05$, and *ns* $P > 0.05$.

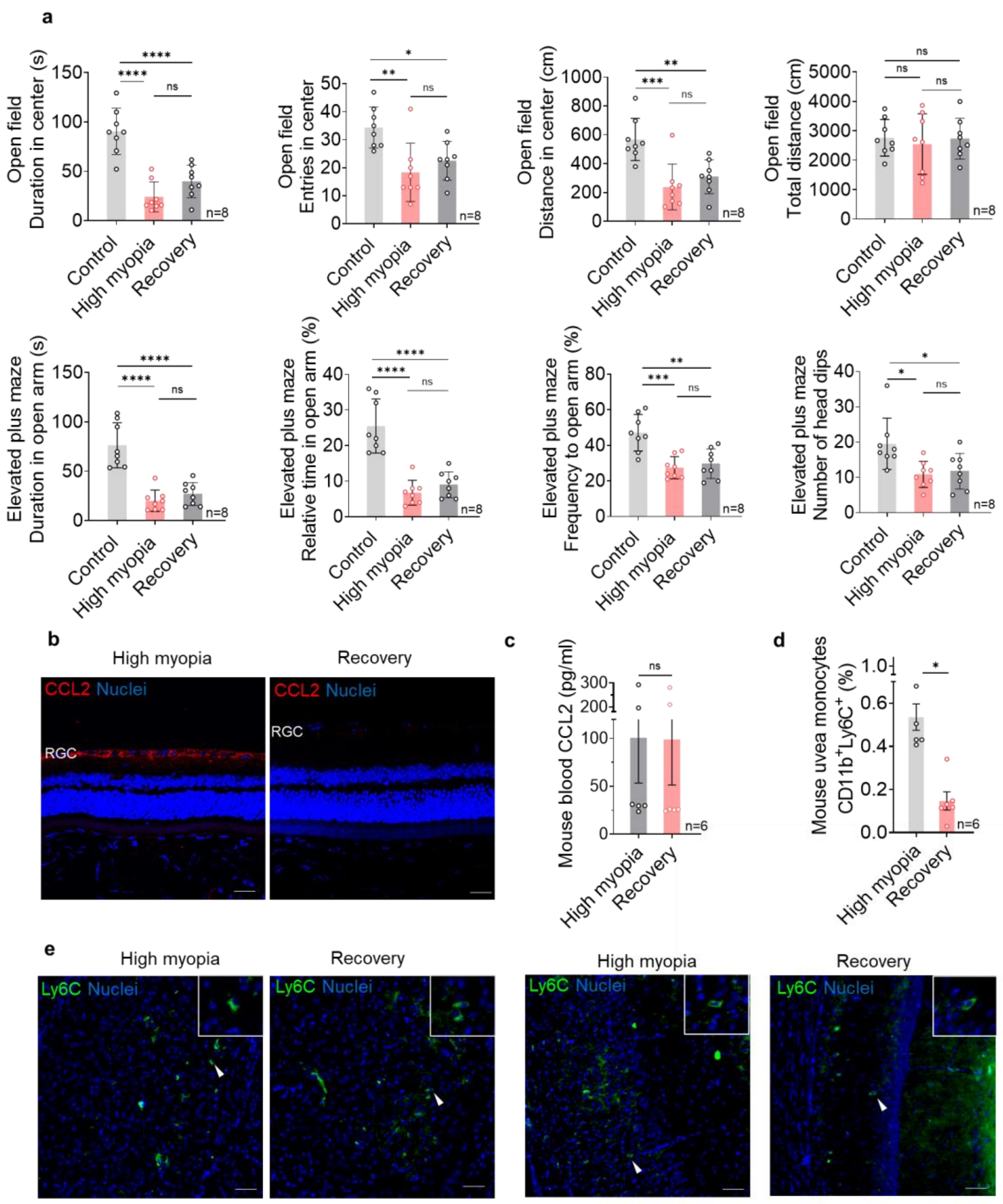
a
Open field
Duration in center (s)
Open field
Entries in center
Open field
Distance in center (cm)
Open field
Total distance (cm)
Elevated plus maze
Duration in open arm (s)
Elevated plus maze
Relative time in open arm (%)
Elevated plus maze
Frequency to open arm (%)
Elevated plus maze
Number of head dips
Control
High myopia
Recovery
n=8
****
***
**
*
ns
b
High myopia
Recovery
CCL2 Nuclei
RGC
c
Mouse blood CCL2 (pg/ml)
n=6
d
Mouse uvea monocytes
CD11b+Ly6C+ (%)
n=6
e
Ly6C Nuclei

Supplementary Fig. S3. The rescue experiment for high myopia. (**a**) Both open field and elevated plus maze tests showed that the anxiety was not reversed by recovery of high myopia at one week after removal of the -10D lens. (**b**) Immunofluorescent images of CCL2 staining in the mouse retina showing downregulated CCL2 expression after recovery of high myopia. Scale bar: 25 µm. (**c**) Measurement of CCL2 concentration in mouse blood. (**d**) Flow-cytometric quantification showing lower ratio of $CD11b^+/Ly6C^+$ monocytes in the mouse uvea after recovery of high myopia. (**e**) Immunofluorescent images showing $Ly6C^+$ monocyte infiltration (arrowheads) into the basal lateral amygdala (BLA) and ventral hippocampus of highly myopic mice was not reduced after recovery of high myopia. Enlarged images in white squares are shown on the top right. Scale bar: 100 µm. (**a**) $n = 8$ in each group. (**b,e**) $n = 3$. (**c,d**) $n = 6$ in each group. Data are means ± SD. Levels of significance were detected with (**a**) one-way ANOVA or (**c,d**) Student's *t* test. ****$P < 0.0001$, ***$P < 0.001$, **$P < 0.01$, *$P < 0.05$, and *ns* $P > 0.05$.

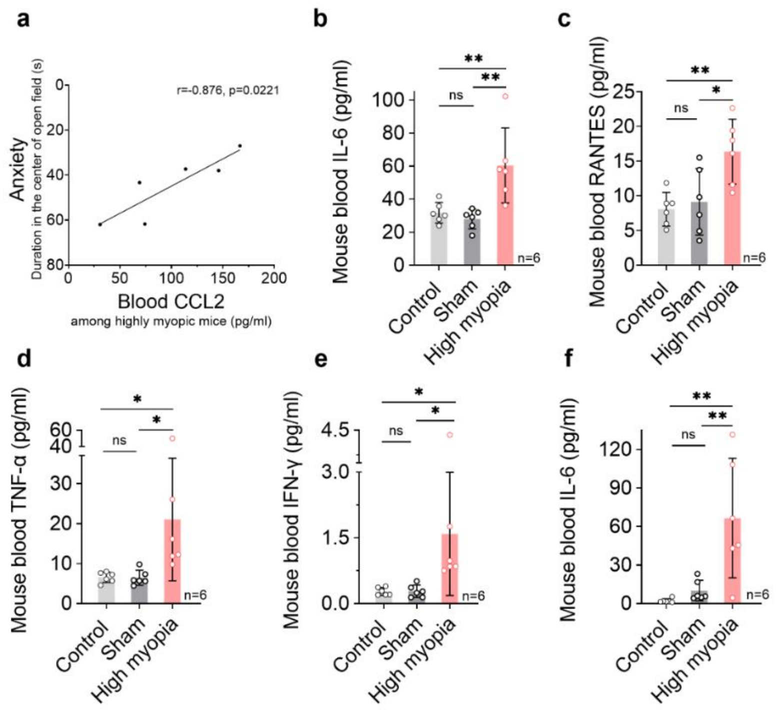

a
r=-0.876, p=0.0221
Anxiety
Duration in the center of open field (s)
Blood CCL2
among highly myopic mice (pg/ml)
b
Mouse blood IL-6 (pg/ml)
c
Mouse blood RANTES (pg/ml)
d
Mouse blood TNF-α (pg/ml)
e
Mouse blood IFN-γ (pg/ml)
f
Mouse blood IL-6 (pg/ml)
Control
Sham
High myopia
ns
n=6

Supplementary Fig. S4. The highly myopic mouse model exhibits elevated levels of inflammatory cytokines in the blood. (**a**) The duration in the center of open field was negatively associated with blood CCL2 level among highly myopic mice. (**b-f**) Measurement of MIP-1β, RANTES, TNF-α, IFN-γ, and IL-6 levels in mouse blood with a proinflammatory biomarker array. Data are means ± SD. n = 6 in each group. Level of significance was detected with (**a**) Pearson's analysis or (**b-f**) one-way ANOVA. ***P* < 0.01, **P* < 0.05, and *ns P >0.05*.

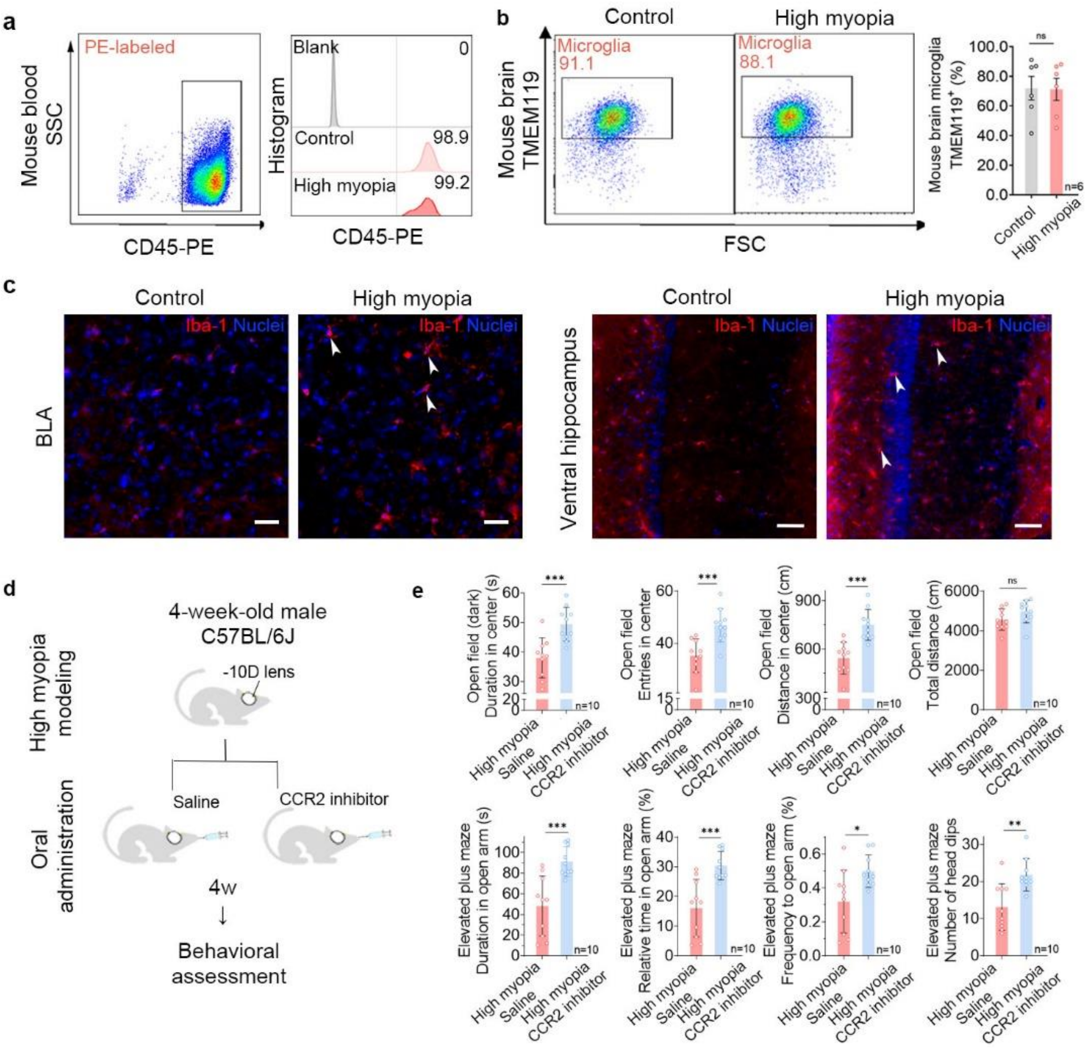
a
PE-labeled
Mouse blood SSC
CD45-PE
Histogram
Blank
0
Control
98.9
High myopia
99.2
CD45-PE
b
Control
High myopia
Mouse brain TMEM119
Microglia
91.1
Microglia
88.1
FSC
Mouse brain microglia TMEM119+ (%)
ns
n=6
Control
High myopia
c
Control
High myopia
Control
High myopia
Iba-1 Nuclei
BLA
Ventral hippocampus
d
4-week-old male C57BL/6J
High myopia modeling
-10D lens
Oral administration
Saline
CCR2 inhibitor
4w
Behavioral assessment
e
Open field (dark) Duration in center (s)
Open field Entries in center
Open field Distance in center (cm)
Open field Total distance (cm)
Elevated plus maze Duration in open arm (s)
Elevated plus maze Relative time in open arm (%)
Elevated plus maze Frequency to open arm (%)
Elevated plus maze Number of head dips
***
***
***
ns
***
***
*
**
n=10
High myopia Saline
High myopia CCR2 inhibitor

Supplementary Fig. S5. The role of brain microglia and infiltrated monocytes in the high myopia-related anxiety. (**a**) Flow-cytometric images showing that peripheral immune cells were labeled with PE-conjugated anti-CD45 antibody in both control and high myopic mice. (**b**) Flow-cytometric quantification showing no difference in ratio of microglia (TMEM119^{+}) in the brain of control and highly myopic mice (n = 6 in each group). (**c**) Immunofluorescent images of Iba-1^{+} microglia (arrowheads) in the basal lateral amygdala (BLA) and ventral hippocampus of mice (n = 3). Scale bar: 100 µm. (**d**) Schematic representation of the establishment of highly myopic mouse model with administration of CCR2 inhibitor. (**e**) Administration of CCR2 inhibitor (RS504393) that blocked the monocyte chemotactic activity, substantially attenuated the anxiety-like behaviors in the highly myopic mice in the open field and elevated plus maze tests (n = 10 in each group). Data are means ± SD. Level of significance was detected with two-sided Student's t test. ***P < 0.001, **P < 0.01, *P < 0.05, and *ns P >0.05*.

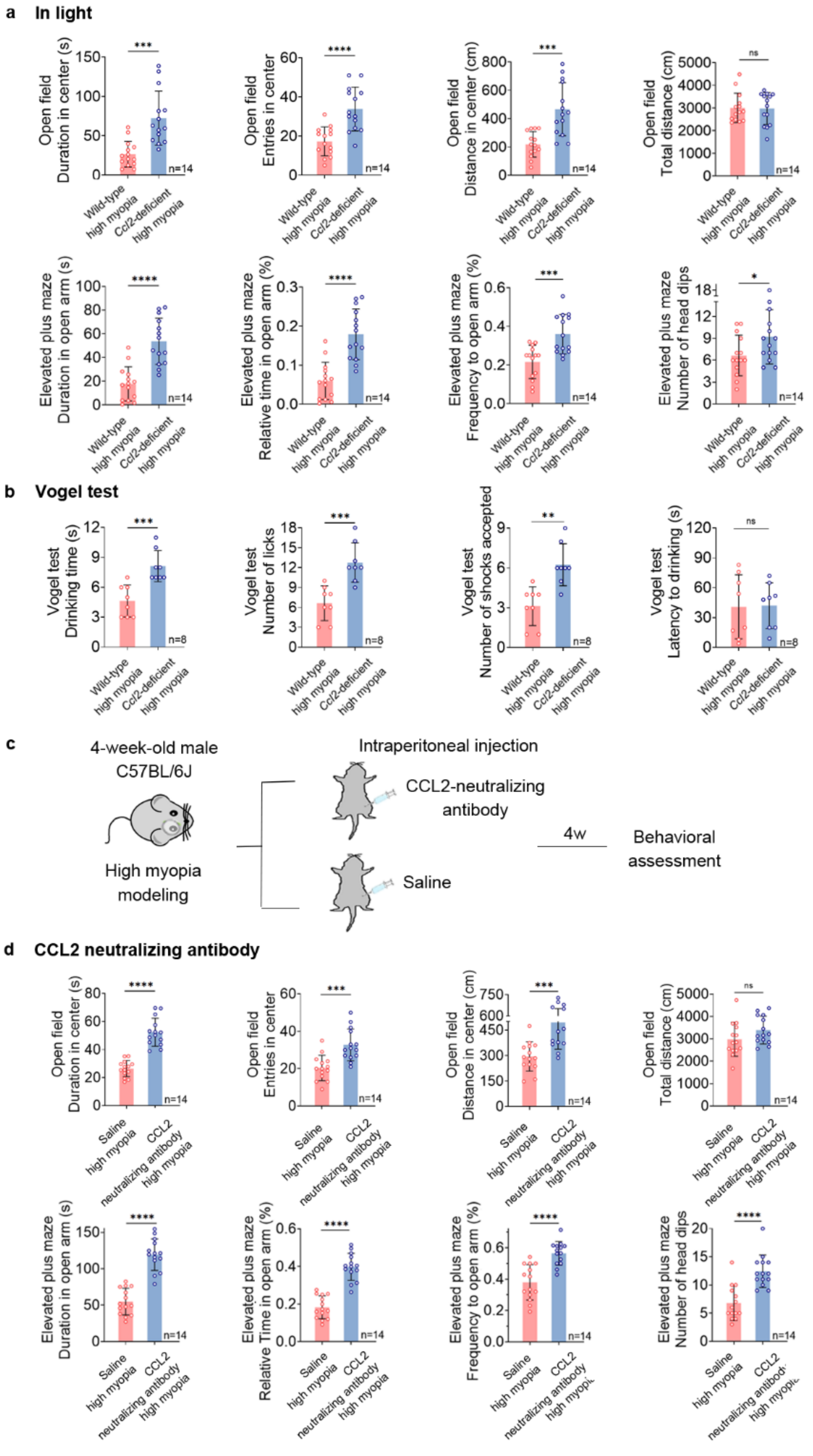
a In light
Open field Duration in center (s)
Open field Entries in center
Open field Distance in center (cm)
Open field Total distance (cm)
Elevated plus maze Duration in open arm (s)
Elevated plus maze Relative time in open arm (%)
Elevated plus maze Frequency to open arm (%)
Elevated plus maze Number of head dips
Wild-type high myopia
Ccl2-deficient high myopia
n=14
***
****
ns
*
b Vogel test
Vogel test Drinking time (s)
Vogel test Number of licks
Vogel test Number of shocks accepted
Vogel test Latency to drinking (s)
n=8
**
c
4-week-old male C57BL/6J
High myopia modeling
Intraperitoneal injection
CCL2-neutralizing antibody
Saline
4w
Behavioral assessment
d CCL2 neutralizing antibody
Open field Duration in center (s)
Open field Entries in center
Open field Distance in center (cm)
Open field Total distance (cm)
Elevated plus maze Duration in open arm (s)
Elevated plus maze Relative Time in open arm (%)
Elevated plus maze Frequency to open arm (%)
Elevated plus maze Number of head dips
Saline high myopia
CCL2 neutralizing antibody high myopia

Supplementary Fig. S6. ***Ccl2*** **deficiency or CCL2 blockage attenuated anxiety in highly myopic mice under different conditions.** (**a**) In light, the *Ccl2*-deficient highly myopic mice exhibited substantially attenuated anxiety than the wild-type highly myopic mice in the open field and elevated plus maze tests. (**b**) Vogel test showed substantially attenuated anxiety-like behaviors in the *Ccl2*-deficient highly myopic mice than the wild-type highly myopic mice. (**c**) Schematic representation of the establishment of highly myopic mouse model with administration of CCL2-neutralizing antibody. (**d**) The highly myopic mice treated with a CCL2-neutralizing antibody exhibited attenuated anxiety than those treated with saline in the open field and elevated plus maze tests. (**a, d**) $n = 14$ in each group; (**b**) $n = 8$ in each group; Level of significance was detected with two-sided Student's t test. $^{****}P < 0.0001$, $^{***}P < 0.001$, $^{**}P < 0.01$, $^{*}P < 0.05$, and *ns* $P > 0.05$.

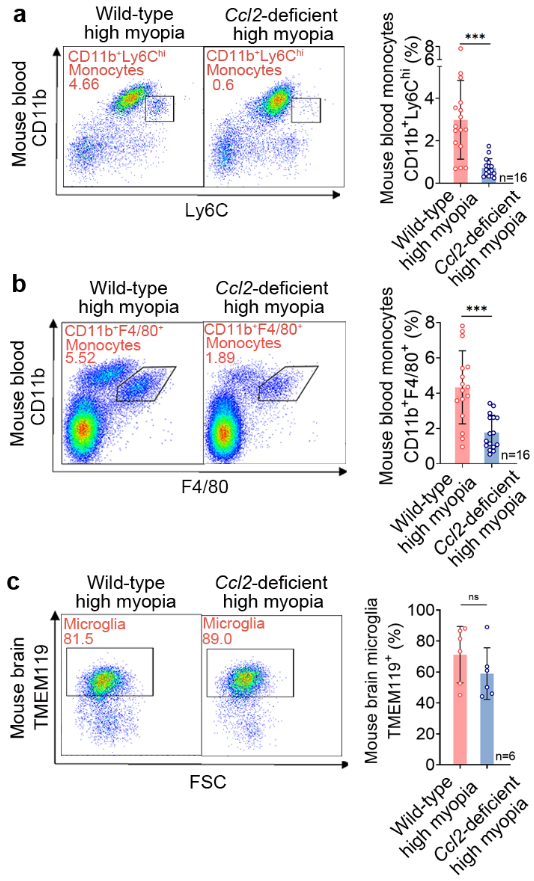

a
Wild-type high myopia
Ccl2-deficient high myopia
CD11b+Ly6Chi Monocytes 4.66
CD11b+Ly6Chi Monocytes 0.6
Mouse blood CD11b
Ly6C
Mouse blood monocytes CD11b+Ly6Chi (%)
***
n=16
b
Wild-type high myopia
Ccl2-deficient high myopia
CD11b+F4/80+ Monocytes 5.52
CD11b+F4/80+ Monocytes 1.89
Mouse blood CD11b
F4/80
Mouse blood monocytes CD11b+F4/80+ (%)
***
n=16
c
Wild-type high myopia
Ccl2-deficient high myopia
Microglia 81.5
Microglia 89.0
Mouse brain TMEM119
FSC
Mouse brain microglia TMEM119+ (%)
ns
n=6

Supplementary Fig. S7. *Ccl2* deficiency reduces monocyte level in the blood and has no effect on number of microglia in the brain of the highly myopic mice. (**a, b**) Flow-cytometric quantification showing decreased ratios of (**a**) $CD11b^+/Ly6C^{hi}$ monocytes and (**b**) $CD11b^+/F4/80^+$ monocytes in the blood of *Ccl2*-deficient highly myopic mice than in the blood of wild-type highly myopic mice (n = 16 in each group). (**c**) Flow-cytometric quantification showing no difference in the ratio of brain microglia ($TMEM119^+$) between the *Ccl2*-deficient highly myopic mice and wild-type highly myopic mice (n = 6 in each group). Data are means ± SD. Level of significance was detected with two-sided Student's t test. ***$P < 0.001$, *ns* $P > 0.05$.

Supplementary Table

Supplementary Table S1. Demographic and clinical characteristics

	Control (n=106)	High myopia (n=104)	P value
Axial length, mm [a]	23.4 ± 0.8	29.3 ± 2.1	<0.001
Age, years [a]	63.8 ± 8.1	61.6 ± 8.8	0.069
Sex, female/male [b]	65/41	58/46	0.414
Body mass index, kg/m^2 [a]	24.3 ± 3.2	23.6 ± 2.6	0.079
Smoking, N(%) [b]	4(3.8%)	4(3.8%)	0.978
Hypertension, N(%) [b]	35(33%)	25(24%)	0.150
Alcohol consumption, N(%) [b]	3(2.8%)	5(4.8%)	0.454
Occupation type, brain power/physical power [b]	62/44	57/47	0.590
Educational degree, below bachelor/bachelor or above [b]	71/35	65/39	0.497

[a] Student's *t* test.

[b] Chi-square test.
